# Incidence of non-invasive all-cause pneumonia in children in the United States before and after the introduction of pneumococcal conjugate vaccines: a retrospective claims database analysis

**DOI:** 10.1186/s41479-023-00109-5

**Published:** 2023-04-05

**Authors:** Tianyan Hu, Eric M. Sarpong, Yan Song, Nicolae Done, Qing Liu, Esteban Lemus-Wirtz, James Signorovitch, Salini Mohanty, Thomas Weiss

**Affiliations:** 1 Merck & Co., Inc., 126 East Lincoln Ave., Rahway, NJ 07065 USA; 2 Analysis Group, Inc., 111 Huntington Ave, Floor 14, Boston, MA 02199 USA

**Keywords:** Claims database study, Incidence rates, Pediatric pneumonia, PCV7, PCV13, Pneumococcal conjugate vaccines

## Abstract

**Background:**

Pneumonia is the most serious form of acute respiratory infection and *Streptococcus pneumoniae* is a leading cause of pediatric bacterial pneumonia. Pneumococcal conjugate vaccines were introduced in the United States (US) in 2000 (7-valent [PCV7]) and 2010 (13-valent [PCV13]). This study estimated annual incidence rates (IRs) of all-cause pneumonia (ACP) among US children aged < 18 years before and after the introduction of PCV7 and PCV13.

**Methods:**

ACP episodes were identified in the IBM MarketScan Commercial and Medicaid Databases using diagnosis codes. Annual IRs were calculated overall and by inpatient and outpatient settings as the number of episodes per 100,000 person-years (PY) for all children aged < 18 years and by age group (< 2, 2–4, and 5–17 years). National estimates of annual pneumonia IRs were extrapolated using Census Bureau data. Interrupted time series (ITS) analyses were used to assess immediate and gradual changes in monthly pneumonia IRs, adjusting for seasonality.

**Results:**

In the commercially-insured population, ACP IRs declined between the pre-PCV7 period (1998–1999) and late PCV13 period (2014–2018) from 5,322 to 3,471 episodes per 100,000 PY for children aged < 2 years, from 4,012 to 3,794 episodes per 100,000 PY in children aged 2–4 years but increased slightly from 1,383 to 1,475 episodes per 100,000 PY in children aged 5–17 years. The ITS analyses indicated significant decreases in monthly ACP IRs in the early PCV7 period (2001–2005) among younger children and in the early PCV13 period (2011–2013) among all children. Increases were observed in the late PCV7 period (2006–2009) among all age groups, but were only significant among older children. IRs of inpatient ACP decreased across all age groups, but outpatient pneumonia IRs remained stable during the study timeframe, even increasing slightly in children aged 5–17 years. More prominent declines were observed for Medicaid-insured children across all age groups; however, Medicaid IRs were higher than IRs of commercially-insured children during the entire study timeframe.

**Conclusions:**

ACP disease burden remains high in US children of all ages despite overall reductions in incidence rates during 1998–2018 following the introduction of PCV7 and PCV13.

**Supplementary Information:**

The online version contains supplementary material available at 10.1186/s41479-023-00109-5.

## Background

Pneumonia is an acute respiratory infection causing considerable pediatric morbidity and mortality worldwide [1–3]. Globally, there were an estimated 138 million cases of pneumonia in children under age 5 in 2015 [2], with pneumonia accounting for an estimated 14% of all deaths in this age group [4]. Pneumonia also contributes to high healthcare resource utilization [3, 5], with approximately half of children under age 5 years with severe community-acquired pneumonia in developing countries requiring hospitalization [2]. However, the incidence varies widely by geography, with higher rates in developing countries [6].

Among several recognized pathogens, *Streptococcus pneumoniae (S. pneumoniae)* is a leading cause of bacterial pneumonia in children after the first few weeks of life (i.e., pneumococcal pneumonia) [7, 8]. The first pneumococcal conjugate vaccine (PCV), protecting against 7 pneumococcal serotypes (PCV7; serotypes 4, 6B, 9V, 14, 18C, 19F, and 23F), was approved by the United States (US) Food and Drug Administration in 2000, and thereafter recommended by the Advisory Committee on Immunization Practices (ACIP) to be included in the pediatric vaccine schedule for all children younger than two years, and for unvaccinated children between 24 and 59 months old who were at high risk for pneumococcal infections [9]. According to the US vaccination schedule, children should receive four doses (at ages 2, 4, and 6 months, and a booster dose between 12 and 15 months) [10]. By 2008, PCV7’s vaccination coverage among US infants and children was > 90% for at least 3 doses and > 80% for 4 doses [11]. In 2010, the 13-valent PCV (PCV13) with an additional six serotypes (i.e., 1, 3, 5, 6A, 19A and 7F) replaced PCV7 in the US pediatric vaccine schedule, with the same dosing schedule. Vaccination coverage with PCV13 remained at > 90% for at least 3 doses and > 80% for 4 doses throughout 2013–2017 [11].

According to systematic reviews of clinical trials for PCVs, vaccination decreases the incidence of all-cause, radiographically confirmed pneumonia and clinically diagnosed pneumococcal pneumonia among children [12–14]. Moreover, multiple analyses of real-world data have revealed significant reductions in the incidence of ACP-related hospitalizations after the introduction of PCV7, generally exceeding initial expectations based on pre-licensure trials [15–20]. Several studies have documented reductions in the burden of invasive and non-invasive pneumonia in children, including pneumococcal pneumonia hospitalizations, after the introduction of PCV13 [21–23].

Evidence regarding the impact of PCVs on the incidence of pneumonia-related outpatient visits is mixed. While the results of some studies have not shown significant declines in ACP outpatient visits when comparing pre- and post-PCV7 periods (i.e., 1994–2007) [24, 25], a real-world claims study by Zhou et al. observed a 41% decline in outpatient visits due to ACP between 1997–2004 [20]. However, no study has comprehensively evaluated the impact of PCV13 introduction on the incidence of pneumonia-related inpatient and outpatient visits among children in the US, particularly during the late PCV13 period.

New vaccines are currently under development for the prevention of pneumococcal disease, including pneumonia, in children [26–28]. These investigational vaccines contain all serotypes in the currently licensed PCV13 as well as additional serotypes. To better understand the current burden of pneumonia and the potential value of new vaccines, it is important to quantify the time trends of ACP following the introduction of PCV7 and PCV13 and the residual burden that remains prior to the introduction of higher valent PCVs. Thus, the objectives of this study were to evaluate the annual incidence of ACP in children aged < 18 years in the US during 1998–2018, the period spanning before and after vaccine introduction, including changes in the incidence rates over time and the current disease burden.

## Methods

### Data source

This study used claims data from the IBM MarketScan® Commercial Claims and Encounters January 1, 1998 to December 31, 2018) and Multi-State Medicaid databases (January 1, 2001 to December 31, 2018). The commercial database contains enrollment eligibility, demographic characteristics, as well as medical, surgical, and prescription drug utilization and expenditure data for approximately 90 million unique individual employees, their spouses, and dependents covered by employer-sponsored private health insurance. The Medicaid database contains similar data for nearly 16 million Medicaid enrollees from 12 states. Both databases contain information on inpatient services, outpatient services, and long-term care services.

### Study design and patient population

This was a retrospective observational study of administrative claims data from US children (aged < 18 years) enrolled in commercial or Medicaid plans at any time during the timeframe of the study (i.e., 1998–2018). Commercially insured children were eligible for inclusion if they had a pneumonia episode, defined using claims with International Classification of Diseases 9/10^th^ Revision, Clinical Modification (ICD-9-CM and ICD-10-CM) codes, during one of the following periods: pre-PCV7 (1998–1999), early PCV7 (2001–2005), late PCV7 (2006–2009), early PCV13 (2011–2013), or late PCV13 (2014–2018). The early PCV7 period was defined as having 5 years given that vaccination coverage rates increased slowly in the first few years after the PCV7 introduction, with the primary series (≥ 3 doses) reaching at least 50% nationally by 2003 [29]. In contrast, the vaccination coverage rates for PCV13, which replaced PCV7 in 2010, stayed at a constant, high level (exceeding 90% for ≥ 3 doses and 80% for ≥ 4 doses) immediately after the vaccine’s approval [29]. Therefore, the early PCV13 period was defined as 2011–2013, allowing for the detection of an immediate impact of PCV13. The years 2000 and 2010, when the PCV7 and PCV13 vaccines were respectively introduced, were considered transitional years and were excluded from the analyses. Medicaid-insured children were eligible for inclusion if they had a pneumonia episode during the early and late PCV7 or early and late PCV13 periods (i.e., 2001–2018).

### Study outcomes

#### Pneumonia episode identification

All-cause, non-invasive pneumonia episodes (i.e., pneumonia due to any bacterial or viral cause) were defined as one or more outpatient and/or inpatient claims with the presence of an ICD-9-CM or ICD-10-CM diagnosis code in the primary positions of inpatient claims and in the primary or secondary positions of outpatient claims. ICD-9-CM codes 480–486 and 487.0, or ICD-10-CM codes J12-J18, J11.0, A22.1, A37.91, B25.0, and B44.0 were used, and claims with codes for invasive disease (i.e., meningitis, septicemia/bacteremia, and bacteremic pneumonia) and empyema were excluded (see Supplemental Tables 1 and 2 [Additional File 1] for descriptions of the codes used). This definition is the most general for pneumonia and consistent with the definition used in previous research [23]. A new episode was defined by a gap of at least 90 days between claims with pneumonia-related diagnoses. The service date of the first claim during each episode was assigned as the index date, which was used to assign episodes to a calendar year. Episodes spanning two different years were assigned to the year in which the index claim occurred (additional information is provided in the Supplemental Methods [Additional File 1]). Episodes containing at least one inpatient claim were categorized as inpatient episodes, while episodes with no inpatient claims were categorized as outpatient episodes.

#### Risk factors

Risk factors that predispose children to pneumococcal diseases were identified in the 6-month period prior to the start of the index episode (i.e., the first episode for each patient in each calendar year). The risk factor definitions were based on the US Centers for Disease Control and Prevention and ACIP’s recommendation for pneumococcal vaccination in high risk children [30, 31].

#### Inpatient case fatality rates

Case fatality rates (CFRs) were calculated for all hospitalized patients with ACP based on patients’ discharge status. The CFRs were calculated as the number of ACP deaths in each period, divided by the number of ACP inpatient discharges in that period, and expressed as a percentage. The 95% confidence intervals (CIs) for the CFRs were calculated using the Wilson score method [32].

### Statistical analysis

The annual incidence rates (IRs) for ACP episode were calculated by dividing the total number of ACP episodes by the total number of person-years (PY) of health plan enrollment for all children in the corresponding calendar year and were expressed as episodes per 100,000 PY. As the exact date of birth was not available in the eligibility files, age was imputed assuming July 1 as the birthdate within each study year. The average IRs were calculated for each of the five time periods listed above, both for the overall population and stratified by age group (< 2 years, 2–4 years, and 5–17 years), for the commercially insured population and for the Medicaid population.

National estimates of annual IRs were calculated via direct standardization of the IRs in the MarketScan population by age, sex, and insurance type (commercial versus Medicaid) using US Census Bureau data for each study year when data on both the commercially insured population and Medicaid population were available, i.e. 2001–2018 (more details are provided in the Supplemental Methods [Additional File 1]).

#### Analyses of time trends

Interrupted time series (ITS) analyses were conducted to assess changes in the temporal trends of IRs before and after the introduction of PCV7 and PCV13. The ITS analyses were performed using generalized linear models with a negative binomial distribution and log link. Specifically, the pneumonia episode counts in each month were modeled in a segmented regression framework, with the number of enrolled children in each month included as an exposure term.

In the models estimated using the commercially insured population, ACP monthly IRs in each of the early PCV7, late PCV7, early PCV13, and late PCV13 periods were compared to the previous period. Separately, ITS analyses were conducted for the Medicaid population between 2006 and 2018. Due to the lack of Medicaid data availability during the pre-PCV7 period, the Medicaid analyses assessed changes in IRs between the early and late PCV13 periods compared to the previous period. Models adjusted for seasonal fluctuation in IRs by using monthly indicators (see Supplemental Methods [Additional File 1] for additional details).

For each period, adjusted incidence rate ratios (IRRs) and 95% CIs were estimated for change in levels (i.e., an immediate change in the IRs from the previous period) and changes in trend (i.e., a gradual yearly change in the current IRs over time compared to the trends in the previous period). Predicted IRs were also obtained from the negative binomial models. All ITS analyses were conducted for children < 18 years, and separately by age groups (< 2, 2–4, and 5–17 years). In addition, using the estimates from the ITS models, adjusted IRRs were estimated comparing the end of each study period to the end of the prior period and to the end of the pre-PCV7 period, respectively, for commercially insured patients. For Medicaid patients, IRRs were estimated comparing the end of each period to the end of the prior period and to the end of the late PCV7 period. These results incorporate both the immediate and gradual changes in ACP IRs estimated during each period. Bootstrapped 95% confidence intervals were obtained using 10,000 replications.

Statistical analyses were conducted using SAS version 9.4 (SAS Institute, Inc., Cary, North Carolina) and R statistical software (R Foundation for Statistical Computing, Vienna, Austria). Statistical significance was defined as *P* < 0.05. Additional details on the specification of the ITS models are provided in the Supplemental Methods (Additional File 1).

#### Exploratory analyses: pneumococcal and unspecified pneumonia

Two exploratory analyses were conducted with alternative definitions of disease manifestations. In the first exploratory analysis, pneumococcal pneumonia (i.e., pneumonia caused by *S. pneumoniae*), was identified using diagnosis codes for pneumococcal pneumonia (ICD-9-CM: 481; ICD-10-CM: J13), or a combination of diagnosis codes for unspecified pneumonia (ICD-9-CM: 482.9, 485, or 486; ICD-10-CM: J15.9, J18.0) and pneumococcal infection (ICD-9-CM: 041.2; ICD-10-CM: B95.3), also excluding codes for invasive disease [23]. This is the definition with the highest specificity and the lowest sensitivity of the definitions used in this study. While PCVs target pneumococcal pneumonia, determining the contribution of pneumococcal pneumonia to the total (i.e., all-cause) burden of pneumonia is difficult due to diagnostic challenges [33]. Current methods to detect bacterial pathogens in non-invasive pediatric pneumonia are extremely limited; most children are unable to produce adequate sputum for culture, and urine antigen detection assays do not reliably distinguish carriage from disease in pediatric populations. Given these diagnostic challenges, pneumococcal pneumonia is expected to be under-coded in claims data.

In the second exploratory analysis, non-invasive pneumonia with identified pneumococcal pathogen as well as those without pathogen attribution but where pneumococcus is known to have a causative role (i.e., unspecified pneumonia) was identified using ICD-9-CM codes 482.9, 485, or 486, or ICD-10-CM codes J15.x, J18.0, or J18.9, without any code for pneumococcal infection. This manifestation represents a subset of ACP episodes, which can be caused by a wide range of bacterial or viral pathogens.

IRs were calculated in the exploratory analyses using similar approaches as used for ACP in the main analysis.

## Results

### Population at risk

Over the time frame of the study, an average of 7.1 million commercially insured children contributed 5.8 million PY at risk each year (Supplemental Table 3 [Additional File 1]), and 4.3 million Medicaid-insured children contributed 3.5 million PY at risk (Supplemental Table 4 [Additional File 1]). The demographic characteristics for the populations at risk by study period and insurance type are shown in Supplemental Table 5 (Additional File 1). Over time, the mean age of commercially insured patients decreased slightly from 9.36 (SD 5.13) in the pre-PCV period to 9.18 (SD 5.16) years in the late PCV13 period. There was a slight increase in the proportion of patients aged < 2 years, and a corresponding slight decrease in the proportion of patients age 2–4 and 5–17 years, respectively. Moreover, there was an increase between the pre-PCV7 and late PCV13 periods in the proportion of patients living in the Northeast (from 15.5% to 17.9%) and West (from 6.0% to 18.7%), and a decrease of patients living in urban areas (from 221.% to 11.4%). Finally, declines in the proportion of patients with fee-for-service (FFS) plans (33.2% to 1.6%) and point-of-service (POS) plans (from 31.3% to 6.9%) was accompanied by increases in the proportions of patients with all other types of plans, including managed care and consumer directed/high deductible health plans. In the Medicaid population, average age increased between the early PCV7 and late PCV13 periods, from 7.90 (SD 5.24) to 8.58 (SD 5.16) years, due to a decline in the proportions of patients < 2 and 2–4 years, respectively. In this population, the substantial declines in the proportions of patients with FFS plans (from 42.7% to 34.5%) and POS plans (from 30.6% to 0.0%) was accompanied by a rise in the proportion of patients with health maintenance organization (HMO) plans (from 26.7% to 65.3%).

### Patient population

#### Demographic characteristics

The demographic characteristics for the commercially insured patient population with ACP across PCV periods are shown in Table 1. The mean age of patients slightly increased over time, from 6.0 years in the early PCV7 period to 6.4 years in the late PCV13 period, while the proportion aged < 2 years decreased over the same timeframe, from 21.6% to 15.7%. Approximately half of patients were male across all periods (i.e., 54.9% in the pre-PCV7 period to 53.0% in the late PCV13 period). The largest proportions of patients were located in the US South (33.5% to 46.8% across all periods) and in urban areas (67.5% to 85.8% across all periods), which is partially a reflection of the health plans whose data are captured in the MarketScan database. While a variety of plan types were represented in the patient sample, in the late PCV13 period more than half of children (56.0%) were covered under Preferred Provider Organization plans.

**Table 1:** Demographic characteristics^a^ of commercially insured patients with ACP by vaccine period (1998–2018)

	**Pre-PCV7**	**Early PCV7**	**Late PCV7**	**Early PCV13**	**Late PCV13**
	**(1998–1999)**	**(2001–2005)**	**(2006–2009)**	**(2011–2013)**	**(2014–2018)**
**Total number of patients**	***N* = 30,530**	***N* = 342,458**	***N* = 649,445**	***N* = 667,230**	***N* = 600,919**
**Age, mean (SD)**^**b**^	6.0 (4.9)	5.8 (4.8)	6.0 (4.7)	6.2 (4.7)	6.4 (4.8)
< 2 years, (%)	21.6	21.0	19.1	16.6	15.7
2–4 years, (%)	26.0	27.7	27.5	27.6	27.0
5–17 years, (%)	52.4	51.3	53.5	55.8	57.2
**Male, (%)**	54.9	54.0	53.9	53.5	53.0
**Region, (%)**					
Northeast	17.2	10.8	12.1	21.0	22.2
North Central	26.1	23.5	27.3	23.9	20.9
South	42.4	41.5	46.8	33.5	38.3
West	5.3	22.6	13.1	18.7	17.4
Missing/unknown	9.0	1.6	0.7	3.0	1.2
**Urbanicity, (%)**					
Urban	67.5	80.3	83.9	84.3	85.8
Rural	23.5	18.2	15.5	12.8	9.8
Missing	9.0	1.5	0.6	2.9	4.4
**Health plan types, (%)**					
FFS	33.8	6.7	1.6	1.0	1.4
EPO	0.5	0.9	0.7	2.7	1.0
HMO	10.0	23.4	16.1	13.2	10.6
POS	30.0	15.6	9.4	6.1	7.2
PPO	25.2	49.6	66.0	61.5	56.0
CDHP	0.0	1.2	2.5	4.4	10.1
HDHP	0.0	0.0	0.4	4.4	10.1
Missing	0.6	2.6	3.2	6.7	3.5

*Abbreviations*: *CDHP* Consumer-directed health plan, *EPO* Exclusive provider organization, *FFS* Fee-for-service, *HDHP* High-deductible health plan, *HMO* Health maintenance organization, *PCV* Pneumococcal conjugate vaccine, *POS* Point of service, *PPO* Preferred provider organization, *SD* Standard deviation

^a^Patients’ demographic characteristics and risk factors were first determined by each calendar year and then combined by PCV periods, assuming each year has distinct patient population. For each calendar year, patients' demographic characteristics were determined at the index episode, which was defined as the first all-cause non-invasive pneumonia episode in the given calendar year

^b^Patients’ month and day of birth was imputed as July 1st for all patients. Age at onset was calculated as the difference between condition start date and imputed birth date. Patients with negative age at onset were included in the age 0–1 year cohort. SDs for age in each vaccine period were calculated using the pooled SD of the samples in relevant years

Demographic characteristics for the Medicaid population with ACP are shown in Supplemental Table 6 (Additional File 1). Medicaid patients were qualitatively younger than commercially insured children and were covered almost exclusively under Health Maintenance Organization or fee-for-service plans in the late PCV13 period.

#### Risk factors

Risk factors for pneumococcal disease among the ACP patients with commercial insurance and Medicaid are shown in Supplemental Tables 7 and 8 (Additional File 1), respectively. The most common risk factors among both commercially insured and Medicaid patients were chronic lung disease including asthma (7.1%-8.4% and 14.3%-16.1%, respectively, across all periods) and cancer and iatrogenic immunosuppression (1.6%-3.3% and 2.3%-9.8%, respectively, across all periods).

### Crude IRs of ACP

#### Overall episodes

**Fig. 1:**
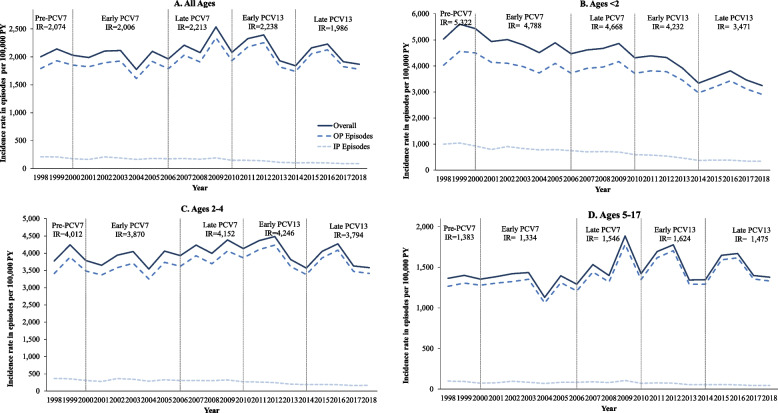
Trends in annual IRs of ACP episodes per 100,000 PY among commercially insured children aged < 18 years (1998–2018). Notes: Average IRs for total ACP episodes are shown for each PCV period. All patients' month and day of birth was imputed as July 1st. Age at onset was calculated as the difference between condition start date and imputed birth date. Patients with negative age at onset were included in the age 0–1 year cohort. Abbreviations: ACP, all-cause pneumonia; IP, inpatient; IR, incidence rate; OP, outpatient; PCV, pneumococcal conjugate vaccine, PY, person-years

The annual IRs for ACP episodes among commercially insured children are presented graphically by age group in Fig. 1a-d, overall and by setting. Average annual IRs by study period and 95% CIs are also presented in Table 2. Among children of all ages, ACP IRs decreased slightly from 2,074 to 1,986 per 100,000 PY between the pre-PCV7 and the late PCV13 period. IRs were highest in the subgroup aged < 2 years and decreased from 5,322 to 3,471 episodes per 100,000 PY from the pre-PCV7 to the late PCV13 period. IRs also decreased slightly in the 2–4-year-old subgroup, from 4,012 to 3,794 episodes per 100,000 PY from the pre-PCV7 to the late PCV13 period. IRs were lowest among the subgroup aged 5–17 years and increased slightly from 1,383 to 1,475 episodes per 100,000 PY in the pre-PCV7 period to the late PCV13 periods, respectively.

**Table 2:** ACP episode IRs and 95% CIs by setting and study period for commercially insured children, in episodes per 100,000 PY, and percent change from prior period (1998–2018)

	**All ages**						**Ages < 2**					
**Period**	**Overall**		**Outpatient**		**Inpatient**		**Overall**		**Outpatient**		**Inpatient**	
	**IR** **(95% CI)**	**%Δ from prior period**	**IR** **(95% CI)**	**%Δ from prior period**	**IR** **(95% CI)**	**%Δ from prior period**	**IR** **(95% CI)**	**%Δ from prior period**	**IR** **(95% CI)**	**%Δ from prior period**	**IR** **(95% CI)**	**%Δ from prior period**
Pre-PCV7	2,074 (2,051; 2,097)	–	1,864 (1,842; 1,885)	–	211 (203; 218)	–	5,322 (5,197; 5,450)	–	4,302 (4,190; 4,417)	–	1,020 (967; 1,077)	–
Early PCV7	2,006 (1,999; 2,013)	-3.3%	1,825 (1,819; 1,832)	-2.1%	181 (179; 183)	-13.8%	4,788 (4,754; 4,823)	-10.0%	3,975 (3,944; 4,007)	-7.6%	813 (799; 828)	-20.3%
Late PCV7	2,213 (2,208; 2,219)	10.3%	2,034 (2,029; 2,039)	11.5%	179 (178; 181)	-1.1%	4,668 (4,642; 4,693)	-2.5%	3,953 (3,929; 3,977)	-0.6%	715 (705; 725)	-12.1%
Early PCV13	2,238 (2,232; 2,243)	1.1%	2,103 (2,098; 2,109)	3.4%	134 (133; 136)	-25.1%	4,232 (4,208; 4,257)	-9.3%	3,700 (3,677; 3,723)	-6.4%	533 (524; 541)	-25.5%
Late PCV13	1,986 (1,981; 1,991)	-11.3%	1,888 (1,883; 1,893)	-10.2%	98 (97; 99)	-26.9%	3,471 (3,449; 3,492)	-18.0%	3,104 (3,083; 3,124)	-16.1%	367 (360; 374)	-31.1%
	**Ages 2–4**						**Ages 5–17**					
	**Overall**		**Outpatient**		**Inpatient**		**Overall**		**Outpatient**		**Inpatient**	
	**IR** **(95% CI)**	**%Δ from prior period**	**IR** **(95% CI)**	**%Δ from prior period**	**IR** **(95% CI)**	**%Δ from prior period**	**IR** **(95% CI)**	**%Δ from prior period**	**IR** **(95% CI)**	**%Δ from prior period**	**IR** **(95% CI)**	**%Δ from prior period**
Pre-PCV7	4,012 (3,927; 4,099)	–	3,652 (3,571; 3,734)	–	361 (336; 387)	–	1,383 (1,362; 1,404)	–	1,287 (1,267; 1,307)	–	96 (91; 102)	–
Early PCV7	3,870 (3,846; 3,894)	-3.5%	3,549 (3,526; 3,572)	-2.8%	321 (314; 328)	-11.1%	1,334 (1,328; 1,340)	-3.5%	1,252 (1,246; 1,258)	-2.7%	82 (80; 83)	-14.6%
Late PCV7	4,152 (4,133; 4,171)	7.3%	3,841 (3,823; 3,859)	8.2%	311 (306; 316)	-3.1%	1,546 (1,541; 1,551)	15.9%	1,455 (1,450; 1,460)	16.2%	90 (89; 92)	11.0%
Early PCV13	4,246 (4,227; 4,265)	2.3%	4,007 (3,989; 4,026)	4.3%	239 (234; 243)	-23.2%	1,624 (1,619; 1,629)	5.0%	1,555 (1,550; 1,560)	6.9%	69 (68; 70)	-24.2%
Late PCV13	3,794 (3,776; 3,812)	-10.6%	3,614 (3,596; 3,631)	-9.8%	180 (176; 184)	-24.7%	1,475 (1,470; 1,480)	-9.2%	1,423 (1,419; 1,428)	-8.5%	51 (50; 52)	-26.1%

95% CIs were calculated using the Pearson method

*Abbreviations*: *ACP* All-cause pneumonia, *CI* Confidence interval, *IR* Incidence rate, *PCV* Pneumococcal conjugate vaccine, *PY* Person-years

**Fig. 2:**
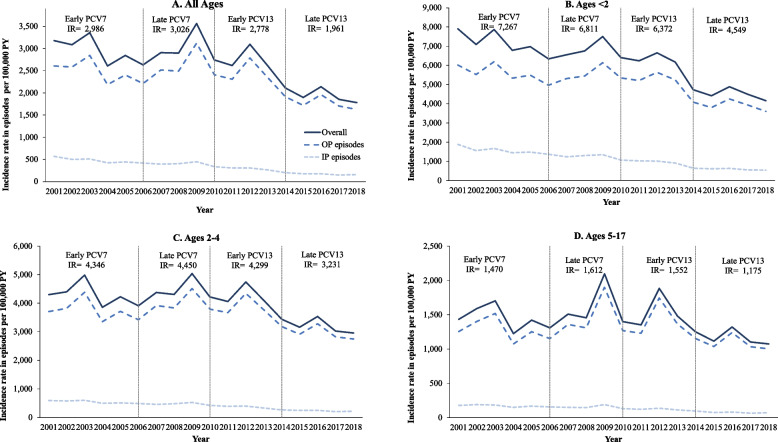
Trends in annual IRs of ACP episodes per 100,000 PY among Medicaid-insured children aged < 18 years (2001–2018). Notes: Average IRs for total ACP episodes are shown for each PCV period. All patients' month and day of birth was imputed as July 1st. Age at onset was calculated as the difference between condition start date and imputed birth date. Patients with negative age at onset were included in the age 0–1 year cohort. Abbreviations: IP, inpatient; IR, incidence rate; OP, outpatient; PCV, pneumococcal conjugate vaccine; PY, person-years

The annual IRs for ACP episodes among children with Medicaid are presented in Fig. 2a-d. Average rates by study period and associated 95% CIs, as well as percent changes from the prior period, are shown in Supplemental Table 9 (Additional File 1). In general, overall IRs were qualitatively higher in Medicaid children compared to commercially insured children, except for the late PCV13 period. Substantial reductions in IRs were observed over the entire study timeframe among Medicaid enrollees of all age groups, including older children. Specifically, ACP IRs decreased between the PCV7 and late PCV13 periods from 7,267 to 4,549 episodes per 100,000 PY among children aged < 2 years, from 4,346 to 3,231 episodes per 100,000 PY among children aged 2–4 years, and from 1,470 to 1,175 episodes per 100,000 PY among children aged 5–17 years. While inpatient episode IRs declined steadily, they remained higher than those in commercially insured children throughout the study timeframe. However, outpatient episode IRs declined rapidly in older Medicaid children, leading to lower levels in the early and late PCV13 periods among the 2–4 and 5–17 year-old groups.

Annual IRs of pneumococcal pneumonia for the commercially insured and Medicaid populations are shown in Supplemental Fig. 1 and Supplemental Fig. 2 (Additional File 1), respectively. Annual IRs of unspecified pneumonia for the commercially insured and Medicaid populations are shown in Supplemental Fig. 3 and Supplemental Fig. 4 (Additional File 1), respectively.

### Inpatient and outpatient episodes

The annual and period-specific IRs of outpatient and inpatient ACP episodes are also shown by age group in Fig. 1a-d, with 95% CIs shown in Table 2. In children of all ages, outpatient episodes comprised more than 80% of ACP episodes among both commercially insured and Medicaid children, and the rates of outpatient episodes exhibited a slight numerical increase over time. When stratified by age groups, outpatient pneumonia IRs decreased substantially in children ages < 2 years, changed little in children aged 2–4 years, and increased in children aged 5–17 years. In contrast, inpatient IRs declined from the pre-PCV period to the late PCV13 period across all age groups. In general, proportions of inpatient episodes among total ACP episodes were numerically higher for younger children compared to older children (results not shown).

Annual and period-specific IRs of outpatient and inpatient pneumonia are shown in Fig. 1a-d for the commercially insured children, and in Fig. 2a-d (with 95% CIs shown in Supplemental Table 9 [Additional File 1]) for Medicaid enrollees. In contrast to the commercially insured population, rates of both outpatient and inpatient episodes decreased over the study timeframe among Medicaid enrollees in all age groups. Despite the downward trends, however, inpatient and outpatient ACP IRs in Medicaid were numerically higher than those in commercially insured children throughout the timeframe of the study, except for the early and late PCV13 periods in older children.

### National IR estimates of ACP

The national estimates of annual IRs of ACP for the overall US population and by age group, along with 95% CIs, are shown in Table 3. The IR for children of all ages decreased from 2,329 to 1,894 episodes per 100,000 PY from 2001 to 2018. By age group, the IRs decreased from 5,761 to 3,649 episodes per 100,000 among children aged < 2 years; from 3,841 to 3,365 episodes per 100,000 PY among those aged 2–4 years; and from 1,452 to 1,284 episodes per 100,000 PY in those aged 5–17 years between 2001 and 2018. Despite the overall decrease in IRs for all age groups, fluctuations in IRs were observed over the course of the study, and IRs peaked in 2009 in all subgroups and in the overall population.

**Table 3:** National estimates for IRs and 95% CIs of ACP, in episodes per 100,000 PY (2001–2018)

Year	All ages		Ages < 2 years		Ages 2–4 years		Ages 5–17 years	
	**IR**	**95% CI**	**IR**	**95% CI**	**IR**	**95% CI**	**IR**	**95% CI**
2001	2,329	(2,307–2,352)	5,761	(5,654–5,870)	3,841	(3,771–3,913)	1,452	(1,433–1,473)
2002	2,397	(2,381–2,412)	5,585	(5,513–5,658)	4,128	(4,076–4,180)	1,507	(1,492–1,521)
2003	2,478	(2,465–2,491)	5,737	(5,679–5,796)	4,364	(4,322–4,407)	1,542	(1,530–1,554)
2004	2,035	(2,024–2,045)	5,196	(5,145–5,246)	3,639	(3,604–3,674)	1,178	(1,169–1,187)
2005	2,352	(2,341–2,363)	5,586	(5,536–5,638)	4,143	(4,108–4,179)	1,441	(1,431–1,451)
2006	2,186	(2,176–2,196)	5,063	(5,020–5,107)	3,986	(3,954–4,019)	1,328	(1,319–1,337)
2007	2,426	(2,416–2,436)	5,261	(5,218–5,305)	4,312	(4,279–4,346)	1,555	(1,545–1,564)
2008	2,312	(2,303–2,322)	5,373	(5,330–5,416)	4,100	(4,069–4,131)	1,429	(1,420–1,438)
2009	2,864	(2,854–2,874)	5,935	(5,892–5,978)	4,646	(4,616–4,677)	1,980	(1,970–1,990)
2010	2,304	(2,295–2,313)	5,134	(5,095–5,172)	4,223	(4,194–4,252)	1,426	(1,418–1,434)
2011	2,418	(2,409–2,426)	5,128	(5,089–5,167)	4,283	(4,255–4,312)	1,570	(1,562–1,578)
2012	2,651	(2,643–2,660)	5,226	(5,189–5,263)	4,594	(4,566–4,621)	1,807	(1,798–1,815)
2013	2,218	(2,210–2,227)	4,905	(4,867–4,944)	3,967	(3,940–3,994)	1,401	(1,393–1,409)
2014	1,982	(1,975–1,989)	3,942	(3,911–3,973)	3,565	(3,541–3,590)	1,315	(1,308–1,322)
2015	2,103	(2,094–2,111)	3,933	(3,897–3,970)	3,713	(3,683–3,743)	1,449	(1,441–1,458)
2016	2,254	(2,245–2,263)	4,239	(4,201–4,278)	3,971	(3,941–4,002)	1,552	(1,543–1,561)
2017	1,955	(1,947–1,964)	3,939	(3,900–3,978)	3,402	(3,373–3,431)	1,316	(1,308–1,325)
2018	1,894	(1,885–1,903)	3,649	(3,612–3,686)	3,365	(3,336–3,395)	1,284	(1,276–1,293)

Rates were obtained by direct standardization of incidence rates in the commercially insured and Medicaid population, by age, sex, and payer, using data from the U.S. Census Bureau, Current Population Survey and Annual Social and Economic Supplements. 95% CIs were calculated using the direct method as outlined by Fay and Feuer (1997) [34]

*Abbreviations: ACP* All-cause pneumonia, *CI* Confidence interval, *IR* Incident rate, *PY* Person-years

### Results of ITS analyses

The estimated IRRs from the ITS analyses of the commercially insured children are shown in Supplemental Table 10, and the monthly predicted IRs of ACP episodes are shown in Supplemental Fig. 1a-d (Additional File 1).

In children aged < 2 years, there was a 52% decrease in monthly ACP IRs by the end of the early PCV7 period (IRR 0.48, 95%CI [0.26, 0.90]), while no statistically significant changes occurred in older children (Table 4). The IRs were relatively unchanged during the late PCV7, such that there was still an overall decline by the end of this period compared to the end of the pre-PCV7 period (IRR 0.53, 95% CI [0.28, 0.99]). The decline resumed during the early PCV13 period (IRR 0.75, 95% CI [0.65, 0.87]), but the IRs stabilized again during the late PCV13 period, with an overall decline of 52% by the end of this period compared to the pre-PCV7 period (IRR 0.48, 95%CI [0.24, 0.94]).

In children aged 2–4 years, the decrease in the early PCV7 period was not statistically significant, and during the late PCV7 period rates were also relatively unchanged. During the early PCV13 period, there was a 20% decline in IRs (IRR 0.80, 95% CI [0.69, 0.92]), with rates down 51% overall since the end of the pre-PCV7 period (IRR 0.49, 95% CI [0.26, 0.91]). Rates increased 31% during the late PCV13 period (IRR 1.31, 95% CI [1.00, 1.75]), and thus the overall change was statistically insignificant compared to the end of the pre-PCV7 period (Table 4).

**Table 4:** Estimated changes in ACP IRs at the end of each PCV study period vs. prior period and pre-PCV7 period among commercially insured children aged 0–17 years (1998–2018)

Period	IRR (95% CI)			
	**All ages**	**Ages < 2 years**	**Ages 2–4 years**	**Ages 5–17 years**
End of Early PCV7 vs. End of Pre-PCV7	0.90 (0.50, 1.62)	0.48 (0.26, 0.90)	0.56 (0.30, 1.02)	1.28 (0.60, 2.72)
End of Late PCV7 vs. End of Early PCV7	1.34 (1.14, 1.55)	1.10 (0.95, 1.27)	1.10 (0.95, 1.28)	1.58 (1.30, 1.92)
End of Late PCV7 vs. End of Pre-PCV7	1.20 (0.67, 2.19)	0.53 (0.28, 0.99)	0.61 (0.33, 1.13)	2.02 (0.95, 4.36)
End of Early PCV13 vs. End of Late PCV7	0.65 (0.56, 0.76)	0.75 (0.65, 0.87)	0.80 (0.69, 0.92)	0.58 (0.48, 0.71)
End of Late PCV7 vs. End of Pre-PCV7	0.78 (0.43, 1.43)	0.40 (0.21, 0.75)	0.49 (0.26, 0.91)	1.18 (0.55, 2.57)
End of Late PCV13 vs. End of Early PCV13	1.51 (1.12, 2.04)	1.19 (0.91, 1.57)	1.31 (1.00, 1.75)	1.68 (1.14, 2.46)
End of Late PCV13 vs. End of Pre-PCV7	1.18 (0.61, 2.31)	0.48 (0.24, 0.94)	0.64 (0.32, 1.26)	1.98 (0.86, 4.70)

[1] Results were calculated using coefficients from a generalized linear model with negative binomial distribution and log link, controlling for seasonality using month indicators

[2] Time periods were defined as follows: Pre-PCV7: 1998–1999; Early PCV7: 2001–2005; Late PCV7: 2006–2009; Early PCV13: 2011–2013; Late PCV13: 2014–2018. Years 2000 and 2010 were considered transition years and were excluded from the model

[3] 95% confidence intervals were obtained by bootstrapping the model coefficients using 10,000 replications

*Abbreviations*: *ACP* All-cause pneumonia, *CI* Confidence interval, *IR* Incidence rate, *IRR* Incidence rate ratio, *PCV* Pneumococcal conjugate vaccine

In children aged 5–17 years, rates did not change significantly during the early PCV7 period, and then increased by 58% during the late PCV7 period (IRR 1.58, 95% CI [1.30, 1.92]). However, IRs declined by 42% during the early PCV13 period (IRR 0.58, 95% CI [0.48, 0.71]). They then increased sharply by 68% during the late PCV13 period (IRR 1.68, 95% CI [1.14, 2.46]), resulting in an insignificant overall change between the end of the pre-PCV7 period and the end of the late PCV13 period.

In the Medicaid population, the findings were qualitatively similar to those in the commercially insured during the early PCV13 period, showing overall declines in monthly ACP IRs in all age groups (Table 5). Specifically, rates declined by 30% among children aged < 2 years (IRR 0.70, 95% CI [0.61, 0.80]), 37% among children aged 2–4 years IRR 0.63, 95% CI [0.54, 0.74]), and 50% among children aged 5–17 years (IRR 0.50, 95% CI [0.40, 0.64]). A key difference, however, is the significant decrease in IRs during the late PCV13 period among all age groups (though only statistically significant among children aged 5–17 years, IRR 0.50, 95% CI [0.32, 0.77]), in contrast to the stabilization or increase in ACP IRs during this period among commercially insured children. The estimates from the ITS models for the Medicaid population are shown in Supplemental Table 11 and the predicted monthly IRs are show in Supplemental Fig. 2 [Additional File 1]).

**Table 5:** Estimated changes in ACP IRs at the end of each PCV study period compared to the prior period and the pre-PCV7 period among Medicaid-insured children aged 0–17 years (2001–2018)

Period	IRR (95% CI)			
	**All ages**	**Ages < 2 years**	**Ages 2–4 years**	**Ages 5–17 years**
End of Early PCV13 vs. End of Late PCV7	0.56 (0.48, 0.66)	0.70 (0.61, 0.80)	0.63 (0.54, 0.74)	0.50 (0.40, 0.64)
End of Late PCV13 vs. End of Early PCV13	0.69 (0.51, 0.93)	0.77 (0.59, 1.01)	0.75 (0.55, 1.01)	0.50 (0.32, 0.77)
End of Late PCV13 vs. End of Pre-PCV13	0.39 (0.28, 0.54)	0.54 (0.41, 0.72)	0.47 (0.34, 0.65)	0.25 (0.16, 0.40)

[1] Results were calculated using coefficients from a generalized linear model with negative binomial distribution and log link, controlling for seasonality using month indicators

[2] Time periods were defined as follows: Late PCV7: 2006–2009; Early PCV13: 2011–2013; Late PCV13: 2014–2018. Year 2010 was considered transition years and was excluded from the model

[3] 95% confidence intervals were obtained by bootstrapping the model coefficients using 10,000 replications

*Abbreviations*: *ACP* All-cause pneumonia, *CI* Confidence interval, *IR* Incidence rate, *IRR* Incidence rate ratio, *PCV* Pneumococcal conjugate vaccine

### Inpatient CFRs

Over the timeframe of the study, inpatient CFRs for commercially insured children with ACP varied between 0.29% and 0.42% in the 0–4 years age group, and between 0.46% and 0.89% in the 5–17 years age group (Table 6). Among Medicaid children, rates were higher, varying between 0.46% and 0.63% for patients 0–4 years old, and between 0.60% and 0.78% for patients 5–17 years old.

**Table 6:** Inpatient CFR and 95% CIs by age group for commercially and Medicaid- insured children aged < 18 years with ACP, by age group (1998–2018)

	**Commercially insured**		**Medicaid**	
Study Period	**Ages 0–4 years**	**Ages 5–17 years**	**Ages 0–4 years**	**Ages 5–17 years**
Pre-PCV7 (1998–1999)	0.30% (0.14%; 0.65%)	0.89% (0.49%; 1.64%)	─	─
Early PCV7 (2001–2005)	0.36% (0.28%; 0.45%)	0.63% (0.49%; 0.79%)	0.46% (0.40%; 0.54%)	0.78% (0.64%; 0.95%)
Late PCV7 (2006–2009)	0.42% (0.36%; 0.50%)	0.52% (0.43%; 0.63%)	0.63% (0.55%; 0.72%)	0.67% (0.53%; 0.85%)
Early PCV13 (2011–2013)	0.35% (0.29%; 0.43%)	0.46% (0.36%; 0.58%)	0.52% (0.44%; 0.63%)	0.72% (0.56%; 0.92%)
Late PCV13 (2014–2018)	0.29% (0.20%; 0.42%)	0.51% (0.36%; 0.72%)	0.53% (0.46%; 0.62%)	0.60% (0.48%; 0.74%)

The 95% confidence intervals were calculated using the Wilson score method [32]

*Abbreviations*: *ACP* All-cause pneumonia, *CI* Confidence interval, *CFR* Case fatality rate, *PCV* Pneumococcal conjugate vaccine

### Exploratory analyses of pneumococcal and unspecified pneumonia

In addition to the more comprehensive, but less specific definition of ACP, we also conducted descriptive exploratory analyses of pneumococcal and unspecified pneumonia IRs. More dramatic declines in the IRs of pneumococcal pneumonia were observed both in commercially insured and Medicaid children across all age groups (Supplemental Figs. 3 and 4 [Additional File 1]). Between 2001 and 2018, pneumococcal pneumonia IRs decreased by approximately 85.4% overall, with the most substantial reduction occurring in children aged < 2 years.

The results of the exploratory analyses on unspecified pneumonia were very similar to theACP results (Supplemental Figs. 5 and 6, respectively [Additional File 1]), suggesting more modest declines over time which occurred mainly in the early PCV13 period and were concentrated largely among younger children.

## Discussion

This study used real-world commercial insurance and Medicaid claims data to examine the IRs of pneumonia in children < 18 years in the US before and after the introduction of PCVs, with a focus on non-invasive ACP. To fill the gap in prior literature, we analyzed non-invasive ACP IRs comprehensively from 1998–2018. The results indicate that the incidence of non-invasive ACP decreased over the study period among children overall (by approximately 20% nationally). However, there was heterogeneity in trends across the commercially insured and Medicaid populations and different age groups. 

More prominent declines in non-invasive ACP were observed during the study timeframe among children aged < 2 years (i.e., 35% among commercially insured children), who were the target population for PCVs. In general, greater reductions in the rates of pneumonia were observed for younger compared to older children. Among commercially insured children in the oldest subgroup, there was a slight increase in non-invasive ACP incidence. In contrast, for children with Medicaid, consistent declines in IRs were observed across all age groups. The results of the ITS analyses suggest that the early PCV7 period was associated with reductions in non-invasive ACP IRs among younger commercially insured children. While the early PCV13 period was associated with gradual reductions in non-invasive ACP IRs, in the late PCV13 period, there were no significant changes in IRs across age groups. Among children with Medicaid, significant declines were observed in the early PCV13 period among the younger age groups, followed by relatively slower declining trends in the late PCV13 period.

In addition, the IRs of inpatient episodes due to non-invasive ACP, representing episodes needing more serious medical attention, steadily declined over the timeframe of the study among all age groups. Similar to the trend for the overall incidence of non-invasive ACP, the declines in inpatient episodes were most pronounced among younger subgroups. 

These results are consistent with studies which have also reported declines in hospitalizations due to pneumonia and large impacts among children < 2 years old [16–20]. For example, Griffin et al. reported in 2013 that following the advent of PCV7, annual pneumonia hospitalization rates in the US (per the Nationwide Inpatient Sample) declined by 551.1 per 100,000 children aged < 2 years, or 47,172 fewer hospitalizations per year in 2009 compared to those expected based on pre-PCV7 rates [16]. Chang et al. examined pneumonia or influenza-related hospitalizations and deaths during 1996–2011 and found declines of 95 and 4.4 per 100,000 people, respectively; children aged < 2 years had the second greatest decline, of 228 per 100,000, behind those aged > 65 years [17]. A time-series analysis by Grijalva et al. reported a decline of 39% in non-invasive ACP admission rates among children aged > 2 years by the end of 2004, or 41,000 pneumonia admissions prevented [18]. Zhou et al. also noted a 52.4% decline in the rate of non-invasive ACP hospitalizations among commercially insured children aged < 2 years following PCV7 introduction [20]. Similarly, a study by Lee et al. using the Kids’ National Database reported that between 1997 and 2006, hospital discharges due to pneumonia substantially decreased among children < 1 year of age [19].

Despite the clear trend in declining pneumonia-related inpatient admissions since the introduction of PCVs, the evidence of similar declines in outpatient pneumonia visits is mixed in both the current study and in the prior literature [20, 24, 25]. In the above-mentioned study by Zhou et al., outpatient visits declined by 41.1% from 1997 to 2004 among commercially insured children aged < 2 years [20]. However, Kronman et al. found no change in the rates of pneumonia-related outpatient visits between 1994 and 2007 among children under < 18 years using National Ambulatory and National Hospital Ambulatory Medical Care Surveys [25]. Another study by Grijalva et al. similarly found no significant decreases in outpatient visit rates for pneumonia among children aged < 2 years [24].

Numerical differences in ACP rates were observed between commercially insured and Medicaid populations over the study timeframe. Non-invasive ACP IRs of Medicaid-insured children were numerically higher than IRs of commercially insured children in all periods except for the late PCV13 period, when Medicaid IRs declined more rapidly and were below commercially insured rates due to the more prominent declines observed in Medicaid IRs. Moreover, outpatient IRs among Medicaid-insured children in the 2–4 year and 5–17 year age groups declined below the IRs among commercially insured children beginning in the early PCV13 period. Although it is unclear what might be causing the observed differences in trends among older Medicaid-insured children, one possibility is that lower barriers to outpatient care access due to spillover effects of the coverage expansions under the Affordable Care Act (ACA) may have led to increased care for preventative services among children [35]. This may have differentially impacted less severe pneumonia cases occurring in older children in Medicaid compared to private plans, while we found that more severe, inpatient cases declined similarly in the two populations. Given the uneven implementation of the ACA in different states and the rotating panel of states included in the MarketScan Medicaid database over time, these findings may be due to sampling bias and should be interpreted with caution [36].

Similarly, persistent differences were observed for case fatality, with CFRs of Medicaid-enrolled children being substantially higher than those of commercially insured children throughout the study timeframe. The CFRs estimated in the current study are largely consistent with those reported in prior literature. For example, Grijalva et al. reported CFRs of between 0.2% and 0.4% in the National Inpatient Sample [18], similar to the rates in the present study of between 0.3% and 0.9%, with some evidence of decline over the study timeframe.

Even with the reductions in ACP IRs observed in this study, there remains a substantial burden of non-invasive ACP. This study found that, in 2018, non-invasive ACP incidence was 1,894 episodes per 100,000 PY overall, with an even higher incidence among younger children. While the integration of PCVs into the US childhood immunization schedule in 2000 has substantially reduced the incidence of pediatric pneumonia, residual disease caused by viral and non-pneumococcal bacterial infections, as well as non-vaccine serotypes and persistent vaccine-type serotypes, remains [37]. Currently, several additional PCVs are under development to further reduce the burden of pneumococcal disease [38]. The investigational PCVs contain all serotypes in the currently licensed PCV13, as well as additional serotypes identified in the interim since the first PCVs, and may provide broader protection. However, the extent to which these PCVs, if approved, will impact non-invasive ACP IRs will depend on the proportion of pneumonia episodes caused by *S. pneumoniae* and by vaccine-type serotypes compared to other viral and bacterial pathogens and non-vaccine serotypes.

The results of the exploratory analyses on pneumococcal pneumonia suggest a stronger relative decline in the IRs compared with the ACP results (including both viral and bacterial pneumonia). This finding is consistent with expectations based on prior PCV efficacy trials [39, 40], given that PCVs specifically target pneumococcal pneumonia. However, most of these declines occurred in the early and late PCV13 periods, whereas the PCV7 periods were associated with more modest declines or even increases among older children. These declines may have occurred because the two vaccines target different serotypes associated with different levels of disease burden [41]. Moreover, another plausible explanation for this finding is the expected delay in the impact of PCV7 in older children due to the lag between vaccination and outcome measurement (e.g., a child vaccinated with PCV7 at age 1 year in 2005 would be included in the 5–17 year subgroup in 2013, which falls in the early PCV13 period). However, this finding may alternatively be an artifact of the challenges of diagnosing pneumococcal pneumonia or may be due to secular changes in diagnosis coding practices. Therefore, care must be taken before drawing strong conclusions based on this finding. Together with the observed trends in outpatient and inpatient ACP episodes, the findings of the sensitivity analysis also suggest that pneumococcal pneumonia may be overrepresented in inpatient episodes, while outpatient pneumonia episodes may be caused by a broader mix of bacterial and viral pathogens.

The results of the exploratory analyses of unspecified pneumonia were largely consistent with those obtained for ACP, indicating declines in IRs mainly in the early PCV13 period and mostly among younger children. This result is also consistent with expectations, given the considerable overlap in diagnosis codes between the unspecified and ACP definitions.

This study is subject to several limitations, some of which are common among retrospective claims database analyses. First, as with any large claims database, miscoding of diagnoses may occur, potentially leading to misclassification and measurement error. Second, pathogen-specific disease episodes caused by *S. pneumoniae* were identified using diagnosis codes; however, lab values for pathogen cultures were not available. Therefore, the IRs of pneumococcal pneumonia may be underestimated. Third, the change from the ICD-9-CM to ICD-10-CM diagnosis coding systems in 2015 may have affected the classification of diseases over time, potentially affecting the comparison of periods before versus after this transition. However, validation studies have suggested that the impact of this change on pneumonia trends over time are minimal [42, 43]. Finally, although the MarketScan database is considered representative of commercial health plans in the US, it is based on non-random sampling. The national estimates of pneumonia IRs therefore rely on the assumption that the study sample accurately reflects the overall commercially insured and Medicaid populations.

## Conclusions

In this study, the IRs of non-invasive ACP among commercially and Medicaid insured children decreased overall during the study period (1998–2018) by approximately 20%, with the highest reduction occurring among children aged < 2 years. Inpatient pneumonia episode rates decreased consistently over the course of the study, while outpatient pneumonia episodes displayed different trends across age groups, with declines in younger children. Despite evidence of substantial declines in IRs of all-cause and pneumococcal pneumonia as a result of PCV vaccination, the burden of pneumonia in US children remains substantial. The extent to which future higher valent PCVs will impact ACP IRs will depend on the proportion of pneumonia episodes caused by *S. pneumoniae* and by vaccine-type serotypes, which should be explored in future real-world studies.

## Supplementary Information

**Additional file 1.**

## Data Availability

The data that support the findings of this study are available from the IBM® MarketScan® Research Databases but restrictions apply to the availability of these data, which were used under license for the current study, and so are not publicly available. Data are however available from the authors upon reasonable request from the corresponding author with permission from IBM® Watson Health™.

## Abbreviations

ACA: Affordable Care Act
ACIP: Advisory Committee on Immunization Practices
ACP: All-cause pneumonia
CFR: Case fatality rate
CI: Confidence interval
ICD-9/10-CM: International Classification of Diseases 9/10^th^ Revision, Clinical Modification
IR: Incidence rate
IRR: Incidence rate ratio
ITS: Interrupted time series
PCV: Pneumococcal conjugate vaccine
PY: Person-years
US: United States
