# Supplementary material for: Incidence of non-invasive all-cause pneumonia in children in the United States before and after the introduction of pneumococcal conjugate vaccines: a retrospective claims database analysis
Source: Pneumonia (Nathan). 2023 Apr 5;15:8. doi: 10.1186/s41479-023-00109-5 (PMC10074783; doi:10.1186/s41479-023-00109-5)
Supplement: Supplementary file 1 — Additional file 1. [file 41479_2023_109_MOESM1_ESM.pdf]

Additional file 1 *for* "Incidence of Non-Invasive All-Cause Pneumonia in Children in the United States before and after the Introduction of Pneumococcal Conjugate Vaccines: A Retrospective Claims Database Analysis"

SUPPLEMENTAL METHODS

Additional information on pneumonia episode definition

Only claims occurring during a patient's enrollment period were included. Claims were excluded if in addition to pneumonia diagnosis codes, they also had diagnosis codes for bacteremia or meningitis, as these conditions were analyzed within the larger research project of which this study was part. This exclusion was performed in order to avoid attributing the health care encounters related to these more severe diseases commonly caused by *S. pneumoniae* to pneumonia. Overall, these exclusions are not likely to substantially affect the rates of pneumonia, given the much higher incidence of pneumonia relative to these diseases.

Derivation of national IR estimates

Data for each study year for which data were available on both the commercially insured and Medicaid populations (i.e., 2001-2018) were obtained from the US Census Bureau database. Estimates of the July 1st US population by sex, age, and insurance type were calculated for each study year by applying the average proportion of individuals aged 0-17 years with private and government health insurance across all age-sex categories. The pneumonia IRs in the general US pediatric population was calculated by multiplying the IRs for each age-sex-insurance type group in the MarketScan data with the proportion of that group in the general US pediatric population and summing the products across all groups. The 95% confidence intervals for the national IR estimates were calculated using the method proposed by Fay and Feuer (1997).

Interrupted time series estimation

Generalized linear models (GLM) with negative binomial family and log link function were used to estimate the parameters of the ITS models. The reasons for using GLM models are that they (1) offer flexibility when the outcome variable is constrained, in our case rates, or when errors are non-normally distributed, (2) are a recommended alternative to a log transformation of the outcome variable and (3) retain a similar analysis framework as linear models. We employed the modified Park test in choosing the appropriate combination of distributional family and link function. The estimating equations used for each of the data sources are shown in detail below.

<u>Equation used for the CCAE models (1998-2018):</u>

$$\ln\left(\mathrm{E}\left[N_i^{AOM}\right]\right) = \ln\left(N_i^{Risk}\right) + \beta_0 + \beta_1 T_i + \beta_2 \times EarlyPCV7_{\mathrm{i}} + \beta_3 \times TEarlyPCV7_{\mathrm{i}} + \beta_4 \times LatePCV7_{\mathrm{i}} + \beta_5 \times TLatePCV7_{\mathrm{i}} + \beta_6 \times EarlyPCV13_{\mathrm{i}} + \beta_7 \times TEarlyPCV13_{\mathrm{i}} + \beta_8 \times LatePCV13_{\mathrm{i}} + \beta_9 \times TLatePCV13_{\mathrm{i}} + \sum_{m=1}^{12} \gamma_m \times I_m$$

where:

N_i^{AOM} is the episode count in each population stratum for month i = 1 to n;

N_i^{Risk} is the population at risk in each population stratum for each month;

T_i is a linear time trend indicator, which equals 1 in January 1998 and increases by 1 with each subsequent month;

$EarlyPCV7$ is an indicator which equals 0 before January 2001 and 1 after the start of the early PCV7 period (i.e. from January 2001 onward);

$TEarlyPCV7$ is a linear time trend indicator for the early PCV7 period, which equals 0 before January 2001, then increases linearly each month as 1, 2, 3, … starting in January 2001;

$LatePCV7$ is an indicator which equals 0 before January 2006 and 1 after the start of the late PCV7 period (i.e. from January 2006 onward);

$TLatePCV7$ is a linear time trend indicator for the late PCV7 period, which equals 0 before January 2006 and then increases linearly each month as 1, 2, 3, … starting in January 2006;

$EarlyPCV13$ is an indicator which equals 0 before January 2011 and 1 after the start of the early PCV13 period (i.e. from January 2011 onward);

$TEarlyPCV13$ is a linear time trend indicator for the early PCV13 period, which equals 0 before January 2011 and then increases linearly each month as 1, 2, 3, … starting in January 2011;

$LatePCV13$ is an indicator which equals 0 before January 2014 and 1 after the start of the late PCV13 period (i.e. from January 2014 onward);

$TLatePCV13$ is a linear time trend indicator for the late PCV13 period, which equals 0 before January 2014 and then increases linearly each month as 1, 2, 3, … starting in January 2014;

I_m is an indicator for each month m = 2 to 12, i.e. February, … December; the indicator for January is excluded and serves as the reference;

<u>Equation used for the Medicaid model (2006-2018):</u>

$$\ln(\mathrm{E}[N_i^{AOM}]) = \ln\left(N_i^{Risk}\right) + \beta_0 + \beta_1 T_i + \beta_2 \times EarlyPCV13_{\mathrm{i}} + \beta_3 \times TEarlyPCV13_{\mathrm{i}} + \beta_4 \times LatePCV13_{\mathrm{i}} + \beta_5 \times TLatePCV13_{\mathrm{i}} + \sum_{m=1}^{12} \gamma_m \times I_m$$

where:

N_i^{AOM} is the episode count in each population stratum for month i = 1 to n;

N_i^{Risk} is the population at risk in each population stratum for each month;

T_i is a linear time trend indicator, which equals 1 in January 2006 and increases by 1 with each subsequent month;

$EarlyPCV13$ is an indicator which equals 0 before January 2011 and 1 after the start of the early PCV13 period (i.e. from January 2011 onward);

$TEarlyPCV13$ is a linear time trend indicator for the early PCV13 period, which equals 0 before January 2011 and then increases each month as 1, 2, 3, … starting in January 2011;

$LatePCV13$ is an indicator which equals 0 before January 2014 and 1 after the start of the late PCV13 period (i.e. from January 2014 onward);

$TLatePCV13$ is a linear time trend indicator for the late PCV13 period, which equals 0 before January 2014 and then increases each month as 1, 2, 3, … starting in January 2014;

I_m is an indicator for each month m = 2 to 12, i.e. February, … December; the indicator for January is excluded and serves as the reference

SUPPLEMENTAL RESULTS

In children <2 years, compared to the pre-PCV7 period, there was an immediate 14.6% decrease (IRR 0.854, 95% CI [0.771 - 0.946], *P*=0.002) in monthly IRs during the early PCV7 period (**Supplemental Table 9**). In the late PCV7 period, there was a 0.3% gradual increase in monthly IRs each month (IRR 1.003, 95% CI [1.000 - 1.006], *P*=0.041).

In children aged 2-4 years, there was a significant gradual increase in monthly IRs of 0.7% each month during the pre-PCV7 period (IRR 1.007, 95% CI [1.004 - 1.010], *P*<0.001). In the early PCV7 period, there was an immediate 14.6% decrease (IRR 0.854, 95% CI [0.768 - 0.951], *P*=0.004) and a gradual decrease of 0.6% each month in monthly IRs (IRR 0.994, 95% CI [0.990 - 0.998], *P*<0.001) compared to the pre-PCV7 period. In the early PCV13 period, there was a gradual decrease of 0.9% each month in monthly IRs (IRR 0.991, 95% CI [0.988 - 0.995], *P*<0.001) compared to the late PCV7 period.

Among children aged 5-17 years, there was a gradual increase in monthly IRs of ACP of 1.1% each month in the late PCV7 compared to the early PCV7 period (IRR 1.011, 95% CI [1.006 - 1.016], *P*<0.001). In the early PCV13 period, a 1.8% gradual decrease (IRR 0.982, 95% CI [0.975 - 0.990], *P*<0.001) occurred in this age group compared to the late PCV7 period.

SUPPLEMENTAL TABLES

Supplemental Table 1. Diagnosis and procedure codes for pneumonia

Condition	Categories	ICD9	ICD10	Descriptions
**Pneumonia**	Pneumococcal specific	481	J13	Pneumococcal pneumonia
		482.9 + 041.2	J15.9 + B95.3	Bacterial pneumonia, unspecified + Pneumococcal infection
		485 + 041.2	J18.0/J18.1 + B95.3	Bronchopneumonia, organism unspecified + Pneumococcal infection
		486 + 041.2	J18.8/J18.9 + B95.3	Pneumonia organism unspecified + Pneumococcal infection
**(without any codes for pneumococcal specific or non-specific meningitis, septicemia, bacteremia, and empyema)**	Unspecified	482.9	J15.9	Bacterial pneumonia, unspecified
		485	J18.0/J18.1	Bronchopneumonia, organism unspecified
		486	J18.8/J18.9	Pneumonia organism unspecified
	All-cause pneumonia	480.x (480.0-480.3, 480.8, 480.9)	J12.x (J12.0, J12.1, J12.2, J12.3, J12.81, J12.89, J12.9)	Viral pneumonia
		481	J13, J18.1	Pneumococcal pneumonia
		482.x	A48.1, J14, J15.0, J15.1, J15.2x (J15.20, J15.211, J15.212, J15.29), J15.3, J15.4, J15.5, J15.6, J15.8, J15.9	Other bacterial pneumonia
		483.x (483.0, 483.1, 483.8)	J15.7, J16.x (J16.0, J16.8)	Pneumonia due to other specified organism
		484.x (484.1, 484.3, 484.5-484.8)	A22.1, A37.X1, B25.0, B44.0, J17	Pneumonia in infectious diseases classified elsewhere
		485	J18.0	Bronchopneumonia, organism unspecified
		486	J18.2, J18.8, J18.9	Pneumonia, organism unspecified
		487.0	J09.X1, J10.0x (J10.00, J10.01, J10.08), J11.0x (J11.00, J11.08)	Influenza with pneumonia

Supplemental Table 2. Diagnosis codes for invasive disease, excluded from the calculation of ACP IR

**IPD**			
**Meningitis**	320.1	G00.1	Pneumococcal meningitis
	320.2+041.2	G00.2+B95.3	Streptococcal meningitis+ Pneumococcal infection
	320.9 +041.2	G00.9+B95.3	Bacterial meningitis, unspecified + Pneumococcal infection
	322.9 +041.2	G03.9+B95.3	Meningitis, unspecified + Pneumococcal infection
**Bacteremia**	038.2	A40.3	Pneumococcal septicemia
	038.0+041.2	A40.9+B95.3	Streptococcal septicemia + Pneumococcal infection
	038.9+041.2	A41.9+B95.3	Unspecified septicemia + Pneumococcal infection
	790.7+041.2	R78.81+B95.3	Bacteremia + Pneumococcal infection
**Bacteremic Pneumonia**	510.x+041.2	J86.x+B95.3	Empyema+ Pneumococcal infection
	513.0+041.2	J85.1+B95.3	Abscess of lung + Pneumococcal infection
	038.2<br>038.0+041.2<br>038.9+041.2<br>790.7+041.2<br>and<br>One code from all-cause pneumonia	A40.3<br>A40.9+B95.3<br>A41.9+B95.3<br>R78.81+B95.3<br>and<br>One code from all-cause pneumonia	One code from the list of pneumococcal specific septicemia and one code from the list of pneumonia
	038.0<br>038.9<br>790.7<br>and<br>481	A40.9<br>A41.9<br>R78.81<br>and<br>J13	
**Other IPD**	--	M00.1x	Pneumococcal arthritis
	567.1	K65.8+B95.3	Pneumococcal peritonitis
	420.9x+041.2	I30.1+B95.3	Infective pericarditis + Pneumococcal infection
	421.0+041.2	I33.0+B95.3	Acute and subacute bacterial endocarditis + Pneumococcal infection
	421.1/421.9 + 041.2	I33.9 +B95.3	Acute and subacute endocarditis, unspecified+ Pneumococcal infection
	567.23+041.2	K65.2+B95.3	Spontaneous bacterial peritonitis+ Pneumococcal infection
	730.0x, 730.2x +041.2	M86.1x/M86.2x/M86.9+B95.3	Acute or unspecified osteomyelitis+ Pneumococcal infection
	711.0x/711.9x +041.2	M00.0x, M00.2x, M00.8x, M00.9 + B95.3	Pyogenic/unspecified arthritis+ Pneumococcal infection

Supplemental Table 3. Commercially insured population at risk, in person-years (1998-2018)

Year	Total Population at risk in person years			
	All ages	Age <2	Age 2-4	Age 5-17
1998	751,826	63,272	102,853	585,701
1999	773,498	64,818	105,906	602,773
2000	822,472	68,020	114,146	640,306
2001	1,231,275	99,473	171,619	960,182
2002	2,440,344	217,813	349,767	1,872,764
2003	3,744,623	335,704	540,081	2,868,838
2004	4,869,883	426,452	704,260	3,739,172
2005	5,336,790	468,088	796,059	4,072,643
2006	6,975,220	633,107	1,014,292	5,327,821
2007	7,157,315	652,795	1,044,293	5,460,227
2008	7,501,516	671,090	1,110,240	5,720,185
2009	8,685,402	774,717	1,328,672	6,582,012
2010	9,763,080	878,663	1,438,974	7,445,443
2011	11,022,990	986,504	1,625,317	8,411,169
2012	10,930,176	941,730	1,598,470	8,389,975
2013	8,845,721	760,576	1,309,439	6,775,706
2014	9,343,998	837,266	1,328,696	7,178,036
2015	5,677,035	510,025	805,890	4,361,120
2016	5,615,207	492,931	800,411	4,321,865
2017	5,253,221	463,883	778,064	4,011,273
2018	5,317,697	487,172	769,136	4,061,389

Notes:

[1] Patients' month and day of birth was imputed as July 1st for all patients. Age at onset was calculated as the difference between condition start date and imputed birth date.

[2] An average of 7.08 million commercially insured children contributed 5.81 million person-years at risk each year

Supplemental Table 4. Medicaid population at risk, in person-years (2001-2018)

	Total Population at risk in person years			
Year	All ages	Age <2	Age 2-4	Age 5-17
2001	1,309,359	236,937	261,527	810,895
2002	2,095,079	360,489	409,664	1,324,926
2003	2,453,468	400,734	483,844	1,568,890
2004	2,581,425	400,834	512,851	1,667,740
2005	2,813,872	436,038	566,058	1,811,776
2006	2,526,832	413,689	483,704	1,629,439
2007	2,406,917	408,134	456,321	1,542,461
2008	2,574,256	430,275	501,395	1,642,585
2009	2,983,346	479,269	614,240	1,889,837
2010	3,139,573	486,189	634,307	2,019,077
2011	2,898,033	426,211	581,710	1,890,111
2012	3,947,665	537,340	779,592	2,630,734
2013	4,032,490	531,923	787,962	2,712,605
2014	5,343,957	712,153	971,404	3,660,400
2015	5,722,235	732,848	1,000,325	3,989,062
2016	5,657,009	684,027	990,031	3,982,951
2017	5,652,255	673,614	1,019,899	3,958,741
2018	4,703,874	577,989	822,895	3,302,990

Notes:

[1] Patients' month and day of birth was imputed as July 1st for all patients. Age at onset was calculated as the difference between condition start date and imputed birth date.

[2] An average of 4.27 million children contributed 3.49 million person-years at risk each year

Supplemental Table 5. Demographic characteristics of the commercially insured and Medicaid population at risk aged <18 by vaccine period (1998-2018)

	Commercially Insured					Medicaid			
	**Pre-PCV7 (1998-1999)**	**Early PCV7 (2001-2005)**	**Late PCV7 (2006-2009)**	**Early PCV13 (2011-2013)**	**Late PCV13 (2014-2018)**	**Early PCV7 (2001-2005)**	**Late PCV7 (2006-2009)**	**Early PCV13 (2011-2013)**	**Late PCV13 (2014-2018)**
**Number of PY at risk**	**1,525,323**	**17,622,916**	**30,319,452**	**30,798,887**	**31,207,158**	**11,253,203**	**10,491,350**	**10,878,188**	**27,079,328**
**Individuals at risk, N**	**1,786,236**	**21,686,073**	**37,053,604**	**37,669,249**	**37,661,189**	**14,686,042**	**13,713,038**	**13,054,994**	**31,476,279**
**Age, mean (SD)**	9.36 (5.13)	9.17 (5.14)	9.10 (5.17)	9.11 (5.13)	9.18 (5.16)	7.90 (5.24)	7.95 (5.30)	8.13 (5.13)	8.58 (5.16)
<2 years, n (%)	128,090 (8.4%)	1,547,530 (8.8%)	2,731,709 (9.0%)	2,688,810 (8.7%)	2,791,277 (8.9%)	1,835,031 (16.3%)	1,731,368 (16.5%)	1,495,474 (13.7%)	3,380,631 (12.5%)
2-4 years, n (%)	208,759 (13.7%)	2,561,786 (14.5%)	4,497,497 (14.8%)	4,533,226 (14.7%)	4,482,198 (14.4%)	2,233,944 (19.9%)	2,055,660 (19.6%)	2,149,265 (19.8%)	4,804,554 (17.7%)
5-17 years, n (%)	1,188,474 (77.9%)	13,513,600 (76.7%)	23,090,246 (76.2%)	23,576,851 (76.6%)	23,933,683 (76.7%)	7,184,228 (63.8%)	6,704,323 (63.9%)	7,233,450 (66.5%)	18,894,144 (69.8%)
**Male, n (%)**	781,982 (51.3%)	9,008,536 (51.1%)	15,486,019 (51.1%)	15,733,677 (51.1%)	15,933,139 (51.1%)	5,730,324 (50.9%)	5,341,057 (50.9%)	5,524,379 (50.8%)	13,847,263 (51.1%)
**Region**									
Northeast	236,547 (15.5%)	1,700,260 (9.6%)	3,280,655 (10.8%)	5,324,554 (17.3%)	5,597,806 (17.9%)	- -	- -	- -	- -
North Central	361,447 (23.7%)	3,855,309 (21.9%)	7,944,535 (26.2%)	7,389,842 (24.0%)	6,640,078 (21.3%)	- -	- -	- -	- -
South	671,600 (44.0%)	7,408,222 (42.0%)	14,189,698 (46.8%)	10,620,846 (34.5%)	12,769,811 (40.9%)	- -	- -	- -	- -
West	91,709 (6.0%)	4,415,513 (25.1%)	4,699,036 (15.5%)	6,651,946 (21.6%)	5,834,866 (18.7%)	- -	- -	- -	- -
Missing/unknown	164,020 (10.8%)	243,612 (1.4%)	205,529 (0.7%)	811,698 (2.6%)	364,597 (1.2%)	- -	- -	- -	- -
**Urbanicity**									
Rural, n (%)	337,203 (22.1%)	3,116,716 (17.7%)	4,802,730 (15.8%)	4,205,925 (13.7%)	3,543,028 (11.4%)	- -	- -	- -	- -
Urban, n (%)	1,023,769 (67.1%)	14,277,841 (81.0%)	25,345,433 (83.6%)	25,792,188 (83.7%)	26,288,879 (84.2%)	- -	- -	- -	- -
Missing	164,351 (10.8%)	228,359 (1.3%)	171,289 (0.6%)	800,774 (2.6%)	1,375,250 (4.4%)	- -	- -	- -	- -
**Health plan types**									
FFS, n (%)	506,524 (33.2%)	1,311,144 (7.4%)	624,969 (2.1%)	311,885 (1.0%)	485,225 (1.6%)	4,800,703 (42.7%)	3,313,709 (31.6%)	3,882,067 (35.7%)	9,330,480 (34.5%)
EPO, n (%)	7,713 (0.5%)	132,809 (0.8%)	216,797 (0.7%)	726,943 (2.4%)	336,806 (1.1%)	0 (0.0%)	0 (0.0%)	0 (0.0%)	0 (0.0%)
HMO, n (%)	133,900 (8.8%)	4,163,711 (23.6%)	4,836,348 (16.0%)	3,963,862 (12.9%)	3,173,868 (10.2%)	3,005,577 (26.7%)	6,174,475 (58.9%)	6,643,672 (61.1%)	17,688,874 (65.3%)
POS, n (%)	477,683 (31.3%)	2,613,797 (14.8%)	2,834,579 (9.3%)	1,904,164 (6.2%)	2,155,477 (6.9%)	3,446,166 (30.6%)	756,055 (7.2%)	343,209 (3.2%)	1,038 (0.0%)
PPO, n (%)	394,230 (25.8%)	8,730,598 (49.5%)	19,809,845 (65.3%)	19,222,249 (62.4%)	17,435,433 (55.9%)	0 (0.0%)	2,794 (0.0%)	0 (0.0%)	37,901 (0.1%)
CDHP, n (%)	0 (0.0%)	214,158 (1.2%)	820,817 (2.7%)	1,471,679 (4.8%)	3,286,131 (10.5%)	0 (0.0%)	0 (0.0%)	0 (0.0%)	0 (0.0%)
HDHP, n (%)	0 (0.0%)	0 (0.0%)	126,870 (0.4%)	1,446,072 (4.7%)	3,219,459 (10.3%)	0 (0.0%)	0 (0.0%)	0 (0.0%)	0 (0.0%)
Missing, n (%)	5,273 (0.3%)	456,699 (2.6%)	1,049,227 (3.5%)	1,752,033 (5.7%)	1,114,759 (3.6%)	757 (0.0%)	244,317 (2.3%)	9,239 (0.1%)	21,036 (0.1%)

Notes:

[1] Patients' month and day of birth was imputed as July 1st for all patients. Age at onset was calculated as the difference between condition start date and imputed birth date.
[2] Patients' demographic characteristics and risk factors were firstly determined by each calendar year and then combined by PCV periods, assuming each year has distinct patient population.
[3] For each calendar year, patients' demographic characteristics were determined at the index episode, which was defined as the first pneumonia episode in the given calendar year.
[4] Standard deviations for age in each vaccine period were calculated using the pooled standard deviation of the samples in relevant years.
[5] All values, except for age and number of individuals at risk are reported in person years. **Abbreviations:** CDHP: Consumer directed health plan; EPO: Exclusive provider organization; FFS: Fee-for-service; HDHP: High-deductible health plan; HMO: Health maintenance organization; PCV: Pneumococcal conjugate vaccine; POS: Point of service; PPO: Preferred provider organization; SD: Standard deviation.

Supplemental Table 6. Demographic characteristics among Medicaid-insured patients aged <18 years with all-cause non-invasive pneumonia by vaccine period (2001-2018)

	Early PCV7 (2001-2005)		Late PCV7 (2006-2009)		Early PCV13 (2011-2013)		Late PCV13 (2014-2018)	
**Total number of patients, N**	**N = 321,484**		**N = 304,134**		**N = 290,443**		**N = 511,939**	
**Age, mean (SD)**	3.84	(4.28)	4.02	(4.27)	4.34	(4.19)	4.73	(4.40)
<2 years, n (%)	127,958	(39.8%)	113,500	(37.3%)	91,628	(31.5%)	148,864	(29.1%)
2-4 years, n (%)	91,695	(28.5%)	86,386	(28.4%)	87,797	(30.2%)	148,084	(28.9%)
5-17 years, n (%)	101,831	(31.7%)	104,248	(34.3%)	111,018	(38.2%)	214,991	(42.0%)
**Male, n (%)**	174,785	(54.4%)	165,483	(54.4%)	155,945	(53.7%)	272,522	(53.2%)
**Health plan types, n (%)**								
EPO	0	(0.0%)	0	(0.0%)	0	(0.0%)	0	(0.0%)
HMO	72,279	(22.5%)	171,009	(56.2%)	167,881	(57.8%)	346,418	(67.7%)
POS	107,052	(33.3%)	23,738	(7.8%)	10,121	(3.5%)	194	(0.0%)
PPO	0	(0.0%)	59	(0.0%)	0	(0.0%)	628	(0.1%)
CDHP	0	(0.0%)	0	(0.0%)	0	(0.0%)	0	(0.0%)
HDHP	0	(0.0%)	0	(0.0%)	0	(0.0%)	0	(0.0%)
FFS	141,947	(44.2%)	100,170	(32.9%)	112,288	(38.7%)	163,880	(32.0%)
Missing	206	(0.1%)	9,158	(3.0%)	153	(0.1%)	819	(0.2%)

Notes:

[1] Patients' month and day of birth was imputed as July 1st for all patients. Age at onset was calculated as the difference between condition start date and imputed birth date. Patients with negative age at onset were included in the analysis.

[2] Patients' demographic characteristics and risk factors were first determined by each calendar year and then combined by PCV periods, assuming each year has distinct patient population.

[3] For each calendar year, patients' demographic characteristics were determined at the index episode, which was defined as the first all-cause non-invasive pneumonia episode in the given calendar year.

[4] Standard deviations for age in each vaccine period were calculated using the pooled standard deviation of the samples in relevant years.

Abbreviations: CDHP: consumer directed health plan; EPO: exclusive provider organization; FFS: fee-for-service; HDHP: high-deductible health plan; HMO: health maintenance organization; PCV: pneumococcal conjugate vaccine; POS: point of service; PPO: preferred provider organization; SD: standard deviation

Supplemental Table 7. Incidence of risk factors for pneumococcal disease among commercially insured ACP patients aged <18 years in the 6 months prior to ACP episodes, by vaccine period (1998-2018)

	Pre-PCV7 (1998-1999)		Early PCV7 (2001-2005)		Late PCV7 (2006-2009)		Early PCV13 (2011-2013)		Late PCV13 (2014-2018)	
**Total number of patients, N**	**N = 24,329**		**N = 238,342**		**N = 498,554**		**N = 450,066**		**N = 495,642**	
**Risk factors**										
Chronic heart disease, n (%)	116	(0.5%)	1,131	(0.5%)	2,427	(0.5%)	2,599	(0.6%)	4,352	(0.9%)
Chronic lung disease including asthma, n (%)	1,729	(7.1%)	17,286	(7.3%)	39,327	(7.9%)	37,837	(8.4%)	41,103	(8.3%)
Diabetes mellitus, n (%)	72	(0.3%)	634	(0.3%)	1,503	(0.3%)	1,395	(0.3%)	2,136	(0.4%)
Cerebrospinal fluid leaks, n (%)	0	(0.0%)	4	(0.0%)	11	(0.0%)	9	(0.0%)	11	(0.0%)
Cochlear implant(s), n (%)	0	(0.0%)	7	(0.0%)	33	(0.0%)	26	(0.0%)	25	(0.0%)
Sickle cell disease or other hemoglobinopathies, and anatomic or functional asplenia, n (%)	66	(0.3%)	544	(0.2%)	1,189	(0.2%)	1,200	(0.3%)	1,155	(0.2%)
Congenital or acquired immunodeficiency, n (%)	43	(0.2%)	496	(0.2%)	1,357	(0.3%)	1,473	(0.3%)	3,255	(0.7%)
HIV infection, n (%)	4	(0.0%)	23	(0.0%)	36	(0.0%)	18	(0.0%)	19	(0.0%)
Chronic renal failure or nephrotic syndrome, n (%)	26	(0.1%)	298	(0.1%)	689	(0.1%)	713	(0.2%)	527	(0.1%)
Cancer and iatrogenic immunosuppression, including radiation therapy, n (%)	801	(3.3%)	3,923	(1.6%)	8,238	(1.7%)	8,930	(2.0%)	12,433	(2.5%)
Solid organ transplant, n (%)	14	(0.1%)	200	(0.1%)	467	(0.1%)	457	(0.1%)	750	(0.2%)
Alcoholism, n(%)	4	(0.0%)	20	(0.0%)	33	(0.0%)	45	(0.0%)	80	(0.0%)
Chronic liver disease, n (%)	18	(0.1%)	107	(0.0%)	278	(0.1%)	279	(0.1%)	625	(0.1%)
Multiple myeloma, n (%)	0	(0.0%)	1	(0.0%)	1	(0.0%)	0	(0.0%)	2	(0.0%)
Tobacco use, n(%)	0	(0.0%)	35	(0.0%)	150	(0.0%)	140	(0.0%)	105	(0.0%)
Hearing loss, n(%)	1	(0.0%)	39	(0.0%)	159	(0.0%)	258	(0.1%)	393	(0.1%)
Pre-term birth, n (%)	46	(0.2%)	600	(0.3%)	940	(0.2%)	622	(0.1%)	1,097	(0.2%)

Notes:
[1] Patients' month and day of birth was imputed as July 1st for all patients. Age at onset was calculated as the difference between condition start date and imputed birth date.
[2] Patients' risk factors were firstly determined by each calendar year and then combined by PCV periods, assuming each year has distinct patient population.
[3] For each calendar year, the index episode was defined as the first all-cause non-invasive pneumonia episode in the given calendar year.
[4] Patients were required to be continuously enrolled in the health plan for at least 6-months prior to the start of index episode.
[5] For each calendar year, risk factors were determined based on medical claims during the 6-months pre-index period.

Abbreviations: PCV: Pneumococcal conjugate vaccine.

Supplemental Table 8. Incidence of risk factors for pneumococcal disease among Medicaid all-cause ACP patients aged <18 years in the 6 months prior to ACP episodes, by vaccine period (2001-2018)

	Early PCV7 (2001-2005)		Late PCV7 (2006-2009)		Early PCV13 (2011-2013)		Late PCV13 (2014-2018)	
**Total number of patients, N**	**N = 218,050**		**N = 219,005**		**N = 225,672**		**N = 430,315**	
**Risk factors**								
Chronic heart disease, n (%)	2,235	(1.0%)	2,412	(1.1%)	2,549	(1.1%)	7,622	(1.8%)
Chronic lung disease including asthma, n (%)	31,229	(14.3%)	32,950	(15.0%)	36,328	(16.1%)	61,539	(14.3%)
Diabetes mellitus, n (%)	798	(0.4%)	838	(0.4%)	861	(0.4%)	2,517	(0.6%)
Cerebrospinal fluid leaks, n (%)	4	(0.0%)	12	(0.0%)	12	(0.0%)	24	(0.0%)
Cochlear implant(s), n (%)	7	(0.0%)	17	(0.0%)	20	(0.0%)	30	(0.0%)
Sickle cell disease or other hemoglobinopathies, and anatomic or functional asplenia, n (%)	2,091	(1.0%)	1,513	(0.7%)	1,635	(0.7%)	3,128	(0.7%)
Congenital or acquired immunodeficiency, n (%)	1,877	(0.9%)	971	(0.4%)	1,016	(0.5%)	4,231	(1.0%)
HIV infection, n (%)	193	(0.1%)	126	(0.1%)	55	(0.0%)	73	(0.0%)
Chronic renal failure or nephrotic syndrome, n (%)	557	(0.3%)	517	(0.2%)	578	(0.3%)	622	(0.1%)
Cancer and iatrogenic immunosuppression, including radiation therapy, n (%)	4,935	(2.3%)	5,588	(2.6%)	14,833	(6.6%)	42,360	(9.8%)
Solid organ transplant, n (%)	1,474	(0.7%)	314	(0.1%)	343	(0.2%)	1,027	(0.2%)
Alcoholism, n(%)	20	(0.0%)	31	(0.0%)	24	(0.0%)	121	(0.0%)
Chronic liver disease, n (%)	259	(0.1%)	223	(0.1%)	230	(0.1%)	975	(0.2%)
Multiple myeloma, n (%)	0	(0.0%)	1	(0.0%)	1	(0.0%)	2	(0.0%)
Tobacco use, n(%)	116	(0.1%)	164	(0.1%)	213	(0.1%)	547	(0.1%)
Hearing loss, n(%)	50	(0.0%)	147	(0.1%)	268	(0.1%)	665	(0.2%)
Pre-term birth, n (%)	1,659	(0.8%)	1,378	(0.6%)	966	(0.4%)	2,787	(0.6%)

Notes:
[1] Patients' month and day of birth was imputed as July 1st for all patients. Age at onset was calculated as the difference between condition start date and imputed birth date.
[2] Patients' risk factors were firstly determined by each calendar year and then combined by PCV periods, assuming each year has distinct patient population.
[3] For each calendar year, the index episode was defined as the first all-cause non-invasive pneumonia episode in the given calendar year.
[4] Patients were required to be continuously enrolled in the health plan for at least 6-months prior to the start of index episode.
[5] For each calendar year, risk factors were determined based on medical claims during the 6-months pre-index period.

Abbreviations: PCV: Pneumococcal conjugate vaccine.

Supplemental Table 9. All-cause pneumonia episode IRs and 95% CIs by setting and study period for Medicaid insured children, in episodes per 100,000 PYs, and percent change from prior period (2001-2018)

Period	All ages						Ages <2					
	Overall		OP		IP		Overall		OP		IP	
	IR (95% CI)	%Δ from prior period	IR (95% CI)	%Δ from prior period	IR (95% CI)	%Δ from prior period	IR (95% CI)	%Δ from prior period	IR (95% CI)	%Δ from prior period	IR (95% CI)	%Δ from prior period
Early PCV7	2,986 (2,976; 2,996)		2,508 (2,499; 2,517)		478 (474; 482)		7,267 (7,228; 7,306)		5,680 (5,646; 5,715)		1,586 (1,568; 1,605)	
Late PCV7	3,026 (3,015; 3,037)	1.3%	2,609 (2,599; 2,619)	4.0%	417 (413; 421)	-12.8%	6,811 (6,772; 6,850)	-6.3%	5,494 (5,459; 5,529)	-3.3%	1,317 (1,300; 1,334)	-17.0%
Early PCV13	2,788 (2,779; 2,798)	-7.9%	2,497 (2,488; 2,507)	-4.3%	291 (288; 295)	-30.2%	6,360 (6,319; 6,400)	-6.6%	5,376 (5,339; 5,413)	-2.1%	984 (968; 1,000)	-25.3%
Late PCV13	1,961 (1,956; 1,967)	-29.7%	1,789 (1,784; 1,794)	-28.4%	172 (171; 174)	-40.9%	4,549 (4,526; 4,572)	-28.5%	3,947 (3,926; 3,968)	-26.6%	602 (594; 610)	-38.8%
	**Ages 2-4**						**Ages 5-17**					
	Overall		OP		IP		Overall		OP		IP	
	IR (95% CI)	%Δ from prior period	IR (95% CI)	%Δ from prior period	IR (95% CI)	%Δ from prior period	IR (95% CI)	%Δ from prior period	IR (95% CI)	%Δ from prior period	IR (95% CI)	%Δ from prior period
Early PCV7	4,346 (4,319; 4,374)		3,797 (3,772; 3,823)		549 (540; 559)		1,470 (1,461; 1,479)		1,297 (1,289; 1,305)		173 (170; 176)	
Late PCV7	4,450 (4,421; 4,479)	2.4%	3,960 (3,933; 3,988)	4.3%	489 (480; 499)	-10.9%	1,612 (1,602; 1,622)	9.7%	1,450 (1,441; 1,459)	11.8%	162 (159; 165)	-6.4%
Early PCV13	4,323 (4,295; 4,351)	-2.9%	3,953 (3,927; 3,980)	-0.2%	370 (362; 378)	-24.3%	1,594 (1,585; 1,603)	-1.1%	1,469 (1,460; 1,478)	1.3%	125 (122; 128)	-22.8%
Late PCV13	3,231 (3,215; 3,247)	-25.3%	2,994 (2,979; 3,010)	-24.3%	237 (232; 241)	-35.9%	1,175 (1,171; 1,180)	-26.3%	1,097 (1,092; 1,102)	-25.3%	79 (77; 80)	-36.8%

Note:
[1] Confidence intervals were calculated using Pearson confidence intervals.

Abbreviations: CI: confidence interval; IP: inpatient; IR: incidence rate; OP: outpatient; PY: person-year.

Supplemental Table 10. Estimates from the interrupted time series analyses of monthly all-cause pneumonia episode IRs in commercially insured children aged <18 years (1998-2018)

Period	IRR	All ages		Ages <2 years		Ages 2-4 years		Ages 5-17 years	
		IRR (95% CI)	*P*	IRR (95% CI)	*P*	IRR (95% CI)	*P*	IRR (95% CI)	*P*
Pre-PCV7	Base Trend	1.001 (0.993 - 1.008)	0.831	1.007 (0.999 - 1.015)	0.079	1.007 (1.004 - 1.010)	< 0.001*	0.996 (0.984 - 1.009)	0.572
Early PCV7	Change in Level	0.984 (0.878 - 1.102)	0.777	0.854 (0.771 - 0.946)	0.002*	0.854 (0.768 - 0.951)	0.004*	1.095 (0.919 - 1.305)	0.311
	Change in Trend	0.999 (0.991 - 1.006)	0.745	0.992 (0.984 - 1.001)	0.066	0.994 (0.990 - 0.998)	< 0.001*	1.002 (0.989 - 1.015)	0.744
Late PCV7	Change in Level	0.934 (0.845 - 1.033)	0.184	0.938 (0.825 - 1.067)	0.330	0.956 (0.853 - 1.071)	0.440	0.934 (0.818 - 1.067)	0.317
	Change in Trend	1.007 (1.003 - 1.011)	< 0.001*	1.003 (1.000 - 1.006)	0.041*	1.003 (0.999 - 1.006)	0.099	1.011 (1.006 - 1.016)	< 0.001*
Early PCV13	Change in Level	1.076 (0.822 - 1.407)	0.594	0.972 (0.812 - 1.164)	0.759	1.100 (0.959 - 1.262)	0.174	1.104 (0.735 - 1.657)	0.635
	Change in Trend	0.986 (0.981 - 0.991)	< 0.001*	0.993 (0.988 - 0.998)	0.004*	0.991 (0.988 - 0.995)	< 0.001*	0.982 (0.975 - 0.990)	< 0.001*
Late PCV13	Change in Level	1.062 (0.905 - 1.247)	0.459	0.930 (0.852 - 1.016)	0.109	1.025 (0.920 - 1.141)	0.655	1.119 (0.907 - 1.381)	0.293
	Change in Trend	1.006 (0.997 - 1.014)	0.172	1.004 (0.999 - 1.010)	0.139	1.004 (1.000 - 1.009)	0.08	1.007 (0.994 - 1.019)	0.288

Notes: All coefficients were obtained through a negative binomial model with a log link, adjusting for seasonality using monthly indicators. IRRs represent the exponentiated regression coefficients and indicate a multiplicative change. Model intercepts are not shown. 95% CIs were adjusted for heteroscedasticity. Abbreviations: CI, confidence interval; IR, incidence rate; IRR, incidence rate ratio; PCV, pneumococcal conjugate vaccine. * $p<0.05$

Supplemental Table 11. Estimates from the ITS analyses of monthly all-cause pneumonia episode IRs in Medicaid children aged <18 years (2006-2018)

		All ages		Ages <2		Ages 2-4		Ages 5-17	
**Period**	**IRR**	**IRR (95% CI)**	**p-value**	**IRR (95% CI)**	**p-value**	**IRR (95% CI)**	**p-value**	**IRR (95% CI)**	**p-value**
Late PCV7	Base Trend	1.008 (1.005 - 1.012)	< 0.001*	1.005 (1.003 - 1.007)	< 0.001*	1.007 (1.005 - 1.010)	< 0.001*	1.012 (1.006 - 1.018)	< 0.001*
Early PCV13	Change in Level	0.795 (0.617 - 1.025)	0.077	0.880 (0.695 - 1.114)	0.288	0.865 (0.682 - 1.098)	0.233	0.678 (0.434 - 1.061)	0.089
	Change in Trend	0.991 (0.983 - 0.999)	0.020*	0.994 (0.988 - 0.999)	0.030*	0.991 (0.985 - 0.998)	0.007*	0.992 (0.979 - 1.004)	0.201
Late PCV13	Change in Level	0.794 (0.674 - 0.935)	0.006*	0.787 (0.714 - 0.867)	< 0.001*	0.838 (0.726 - 0.967)	0.016*	0.756 (0.589 - 0.971)	0.028*
	Change in Trend	0.998 (0.990 - 1.005)	0.556	1.000 (0.994 - 1.006)	0.932	0.998 (0.992 - 1.005)	0.570	0.993 (0.980 - 1.006)	0.298

Notes:
[1] ∗ p<0.05.
[2] All coefficients were obtained through a negative binomial model with a log link. IRRs represent the exponentiated regression coefficients and indicate a multiplicative change. Model intercepts are not shown.
[3] Confidence intervals have been adjusted for heteroscedasticity.
[4] Time periods are defined as follows: Late PCV7: 2006-2009; Early PCV13: 2011-2013; Late PCV13: 2014-2018. Year 2010 is considered a transition year and was excluded from the model.

SUPPLEMENTAL FIGURES

Supplemental Figure 1. Monthly all-cause pneumonia episode IRs predicted from the interrupted time series negative binomial model in the commercially insured population aged <18 years (1998-2018)

Notes:

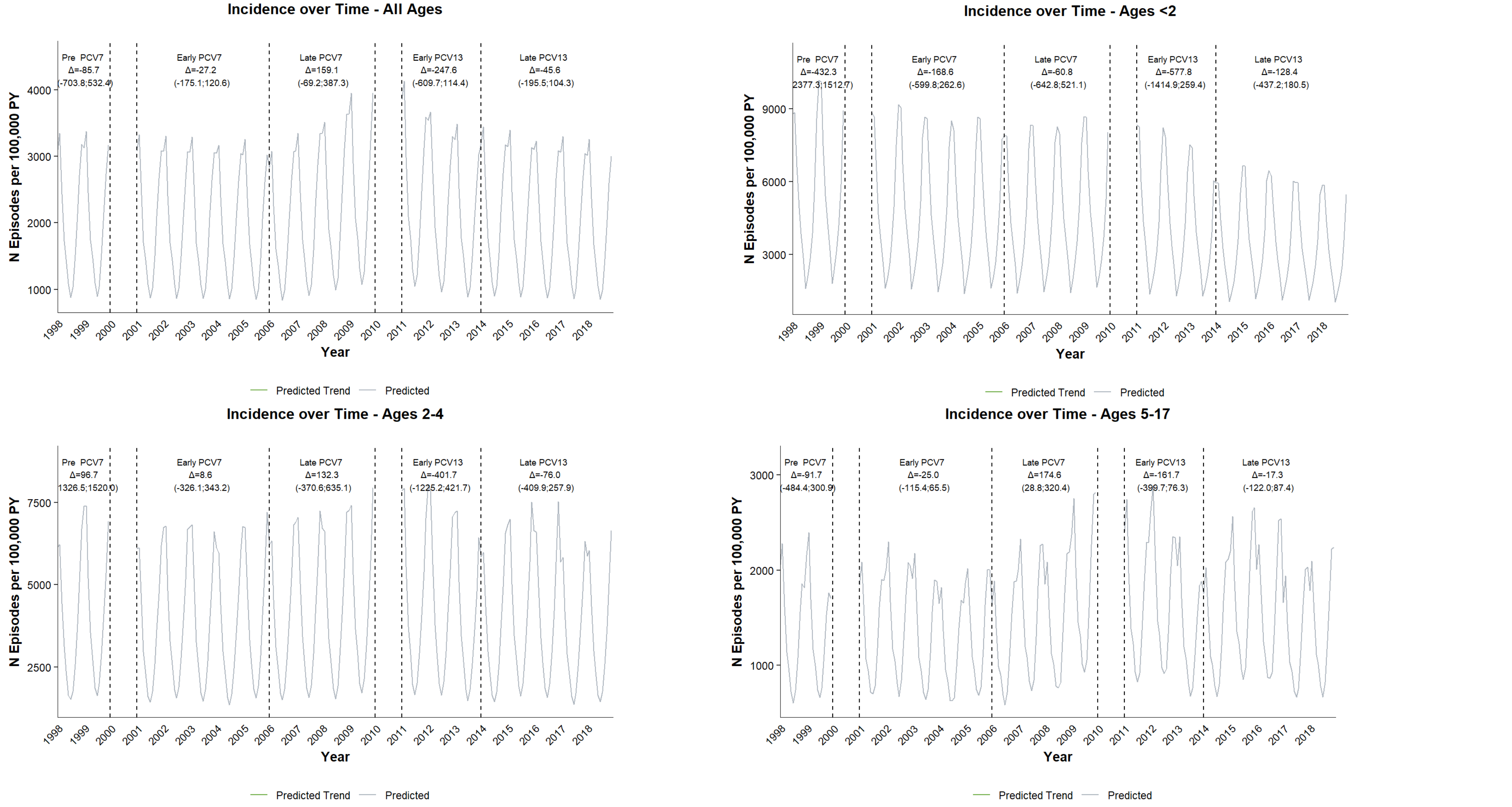

[1] Predicted values for episode counts were obtained using a negative binomial GLM model with a log link.
[2] Time periods are defined as follows: Pre PCV7: 1998-1999; Early PCV7: 2001-2005; Late PCV7: 2006-2009; Early PCV13: 2011-2013; Late PCV13: 2014-2018. Years 2000 and 2010 are considered transition years and were excluded from the model.
Abbreviations: GLM: Generalized linear model; IRs: incidence rates; ITS: Interrupted time series; PCV: Pneumococcal conjugate vaccine; PY: Person-years.

Supplemental Figure 2. Monthly IRs of all-cause pneumonia episodes predicted from the ITS models in Medicaid children aged <18 years (2006-2018)

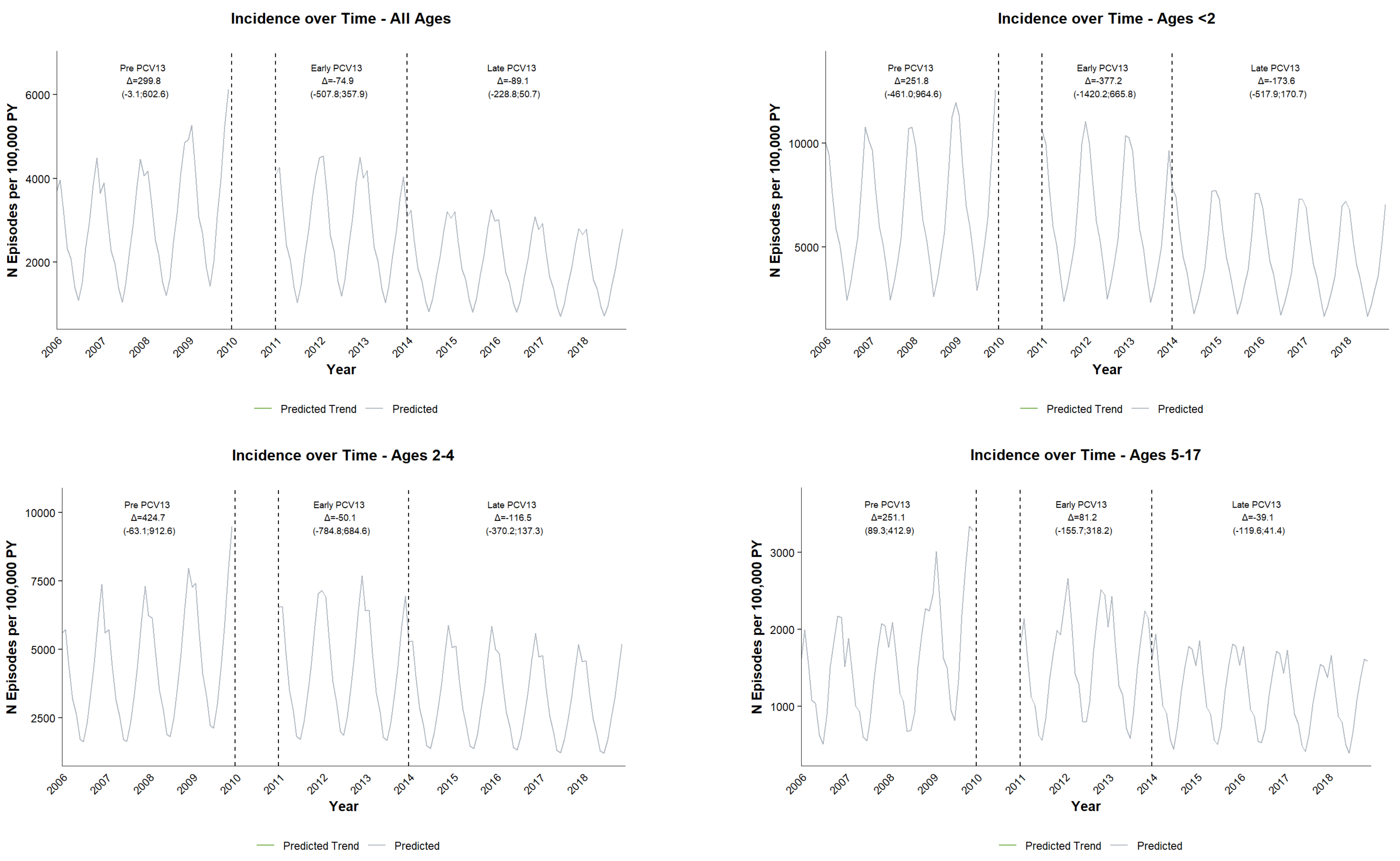

Notes:
[1] Predicted values for episode counts were obtained using a negative binomial GLM model with a log link.
[2] Time periods are defined as follows: Late PCV7: 2006-2009; Early PCV13: 2011-2013; Late PCV13: 2014-2018. The year 2010 is considered a transition year and was excluded from the model. **Abbreviations:** GLM: Generalized linear model; IRs: incidence rates; ITS: Interrupted time series; PCV: Pneumococcal conjugate vaccine; PY: Person-years.

Supplemental Figure 3. Trends in annual incidence rates of pneumococcal non-invasive pneumonia episodes in commercially insured children aged <18 years, in episodes per 100,000 PY (1998-2018)

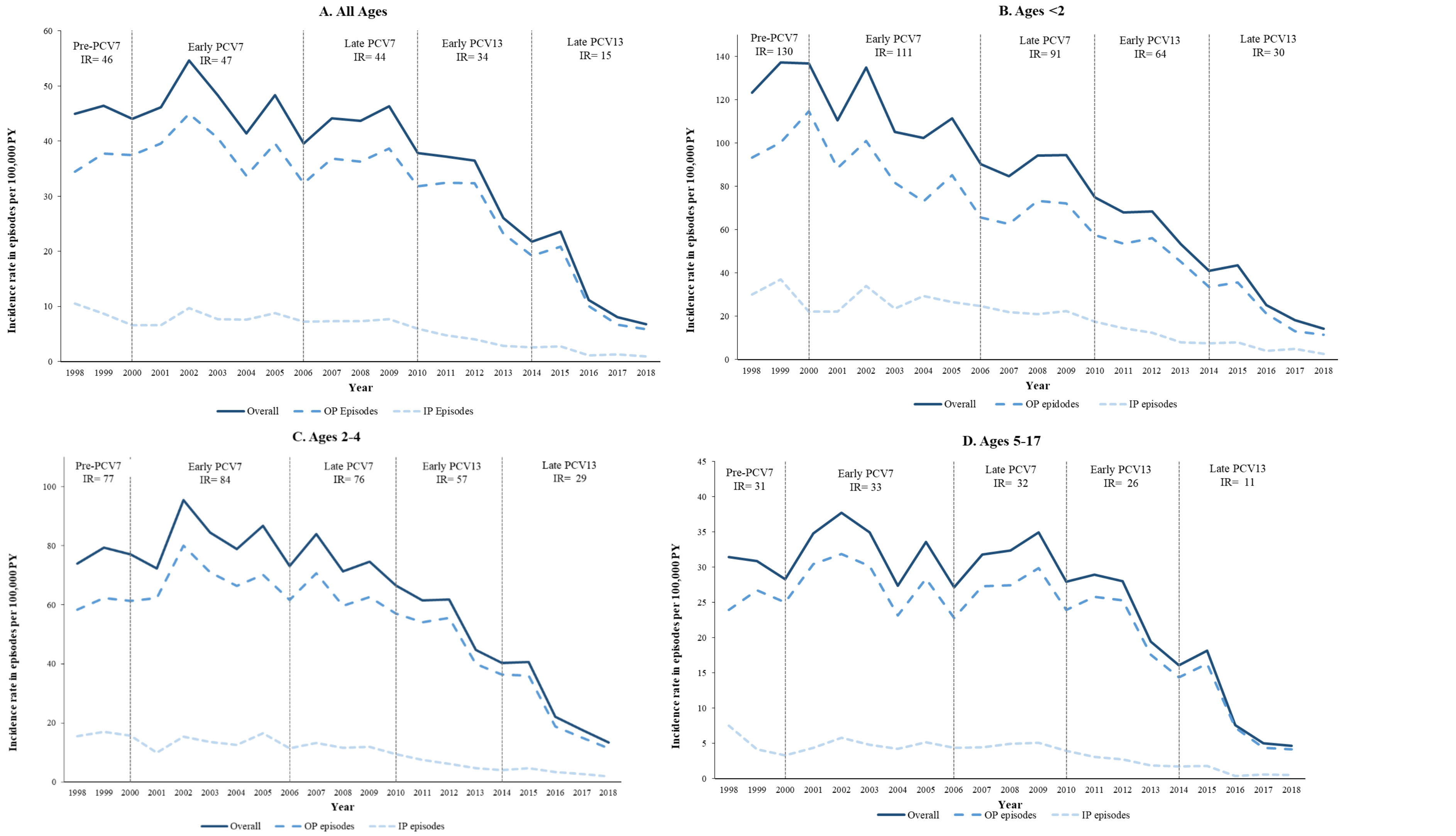

Note:

[1] Average IRs for total pneumococcal non-invasive pneumonia episodes are shown for each of the PCV periods.

Abbreviations: IP: Inpatient; IR: Incidence rate; OP: Outpatient; PCV: Pneumococcal conjugate vaccine, PY: Person-years.

Supplemental Figure 4. Trends in annual IRs of pneumococcal non-invasive pneumonia episodes in Medicaid children aged <18 years (1998-2018)

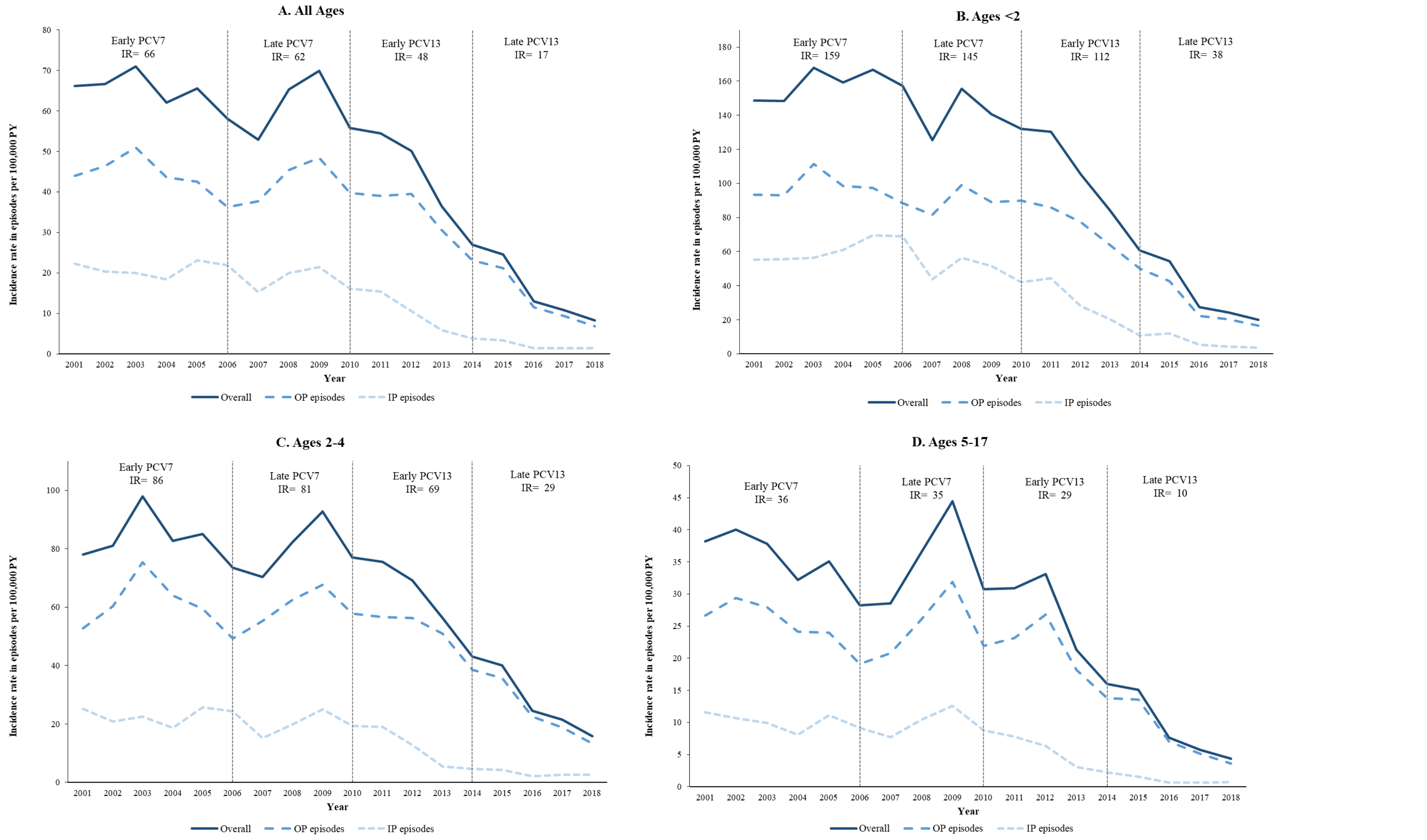

Note:

[1] Patients' month and day of birth was imputed as July 1st for all patients. Age at onset was calculated as the difference between condition start date and imputed birth date. Patients with negative age at onset were included in the age 0-1 cohort.

Abbreviations: IP: Inpatient; IRs: Incidence rates; OP: Outpatient; PCV: Pneumococcal conjugate vaccine, PY: Person-years.

Supplemental Figure 5. Trends in annual IRs of unspecified non-invasive pneumonia in commercially insured children aged <18 years (1998-2018)

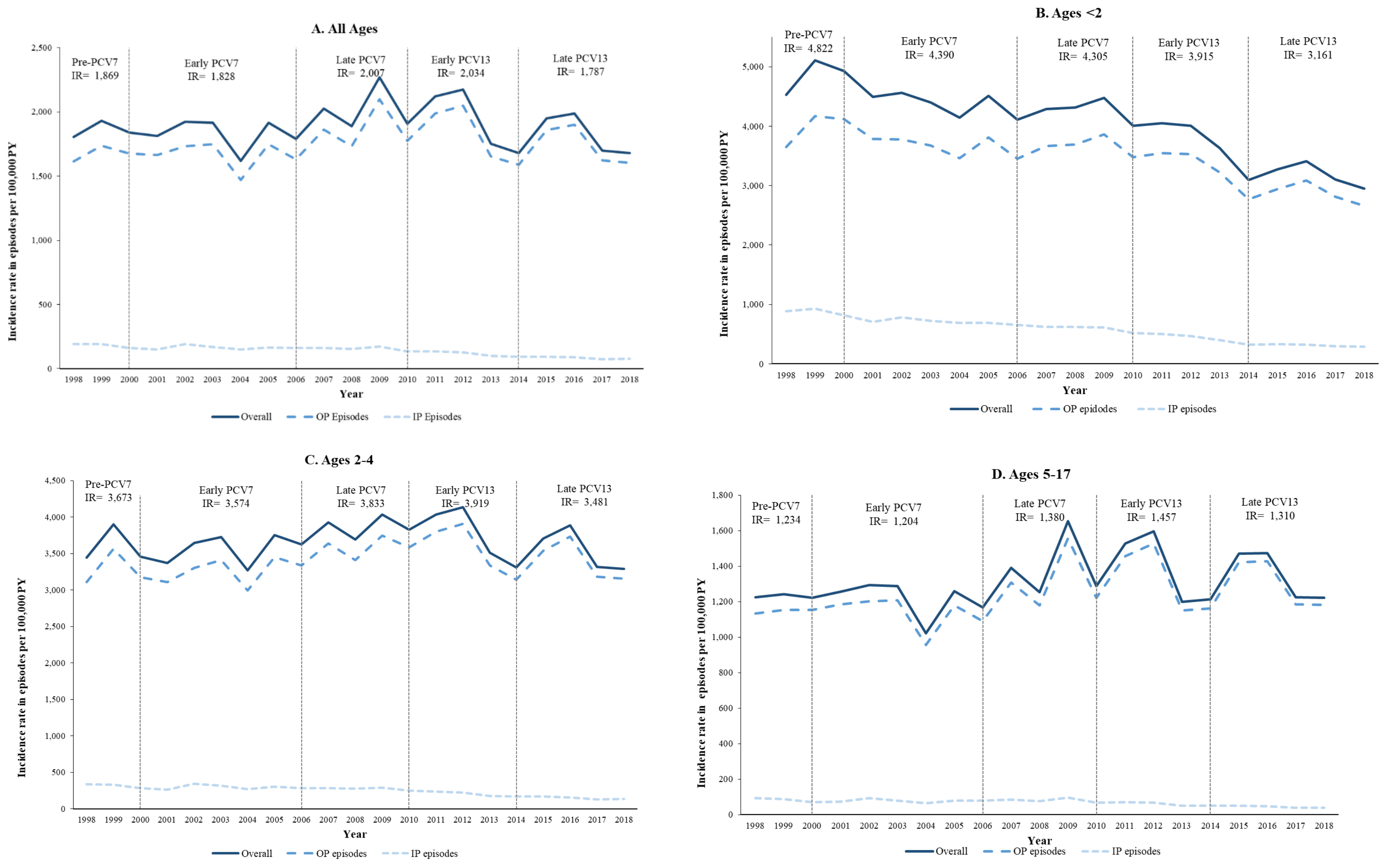

Notes:

[1] Average IRs for total all-cause non-invasive pneumonia episodes are shown for each of the PCV periods.

Abbreviations: IP: Inpatient; IR: Incidence rate; OP: Outpatient; PCV: Pneumococcal conjugate vaccine, PY: Person-years.

Supplemental Figure 6. Trends in annual IRs of unspecified non-invasive pneumonia in Medicaid children aged <18 years (2001-2018)

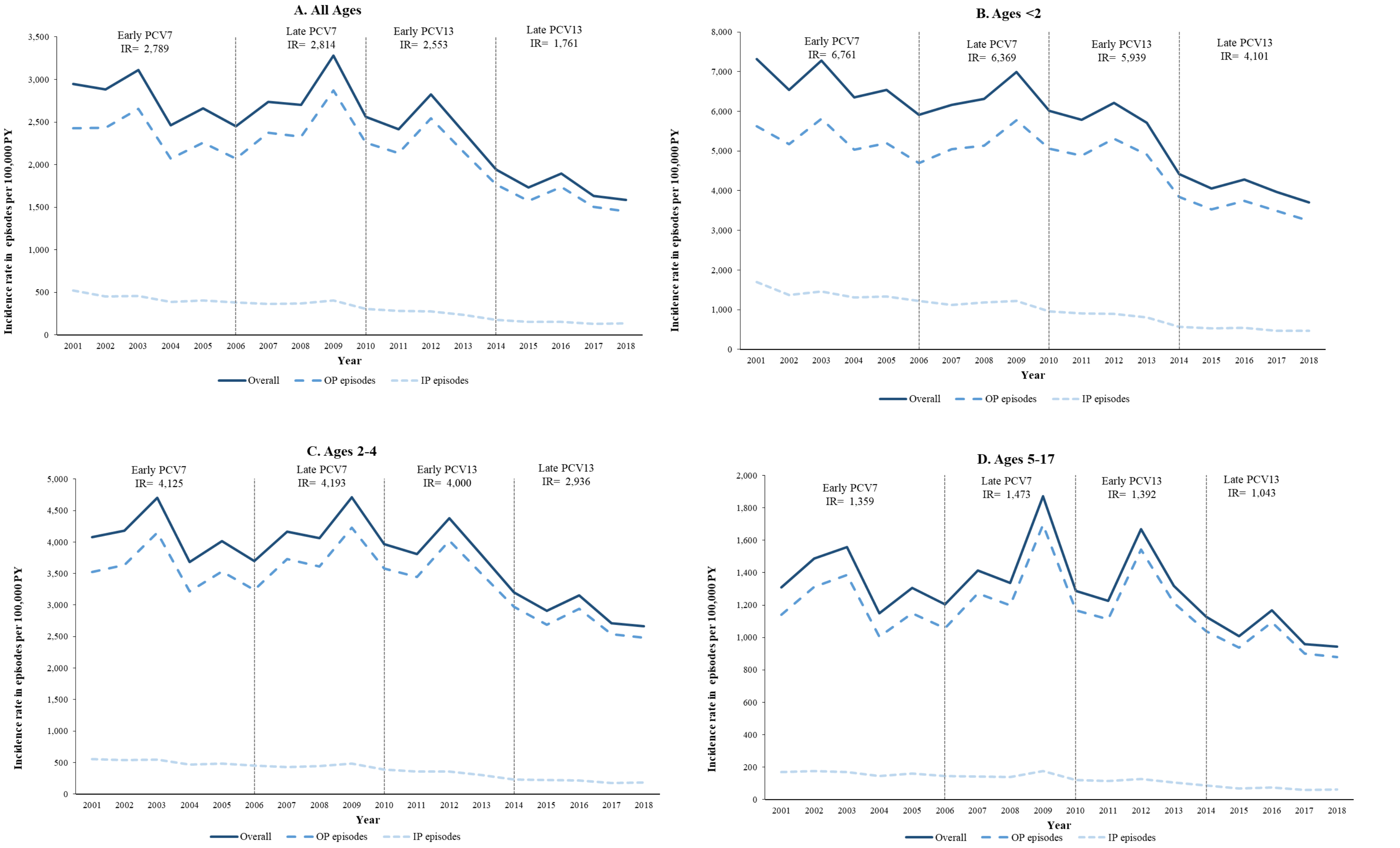

Note:

[1] Patients' month and day of birth was imputed as July 1st for all patients. Age at onset was calculated as the difference between condition start date and imputed birth date. Patients with negative age at onset were included in the age 0-1 cohort.

Abbreviations: IP: Inpatient; IR: Incidence rate; OP: Outpatient; PCV: Pneumococcal conjugate vaccine, PY: Person-years.

References

Fay, M. P. and E. J. Feuer (1997). "Confidence intervals for directly standardized rates: a method based on the gamma distribution." Statistics in medicine **16**(7): 791-801.
